# Reduced detection rate of artificial intelligence in images obtained from untrained endoscope models and improvement using domain adaptation algorithm

**DOI:** 10.3389/fmed.2022.1036974

**Published:** 2022-11-10

**Authors:** Junseok Park, Youngbae Hwang, Hyun Gun Kim, Joon Seong Lee, Jin-Oh Kim, Tae Hee Lee, Seong Ran Jeon, Su Jin Hong, Bong Min Ko, Seokmin Kim

**Affiliations:** ^1^Department of Internal Medicine, Soonchunhyang University College of Medicine, Seoul, South Korea; ^2^Department of Intelligent Systems and Robotics, Chungbuk National University, Cheongju, South Korea

**Keywords:** endoscopes, artificial intelligence, deep learning, generative adversarial network, domain adaptation algorithm

## Abstract

A training dataset that is limited to a specific endoscope model can overfit artificial intelligence (AI) to its unique image characteristics. The performance of the AI may degrade in images of different endoscope model. The domain adaptation algorithm, i.e., the cycle-consistent adversarial network (cycleGAN), can transform the image characteristics into AI-friendly styles. We attempted to confirm the performance degradation of AIs in images of various endoscope models and aimed to improve them using cycleGAN transformation. Two AI models were developed from data of esophagogastroduodenoscopies collected retrospectively over 5 years: one for identifying the endoscope models, Olympus CV-260SL, CV-290 (Olympus, Tokyo, Japan), and PENTAX EPK-i (PENTAX Medical, Tokyo, Japan), and the other for recognizing the esophagogastric junction (EGJ). The AIs were trained using 45,683 standardized images from 1,498 cases and validated on 624 separate cases. Between the two endoscope manufacturers, there was a difference in image characteristics that could be distinguished without error by AI. The accuracy of the AI in recognizing gastroesophageal junction was >0.979 in the same endoscope-examined validation dataset as the training dataset. However, they deteriorated in datasets from different endoscopes. Cycle-consistent adversarial network can successfully convert image characteristics to ameliorate the AI performance. The improvements were statistically significant and greater in datasets from different endoscope manufacturers [original → AI-trained style, increased area under the receiver operating characteristic (ROC) curve, *P*-value: CV-260SL → CV-290, 0.0056, *P* = 0.0106; CV-260SL → EPK-i, 0.0182, *P* = 0.0158; CV-290 → CV-260SL, 0.0134, *P* < 0.0001; CV-290 → EPK-i, 0.0299, *P* = 0.0001; EPK-i → CV-260SL, 0.0215, *P* = 0.0024; and EPK-i → CV-290, 0.0616, *P* < 0.0001]. In conclusion, cycleGAN can transform the diverse image characteristics of endoscope models into an AI-trained style to improve the detection performance of AI.

## 1. Introduction

Deep learning (DL) technology has significantly improved the image recognition capabilities of artificial intelligence (AI) (1). Moreover, lesion detection in endoscopic images using DL-based AI has exhibited remarkable results (2, 3). However, AI performance is significantly influenced by the nature of the data it was trained on. Each endoscopic image exhibits distinct characteristics determined by the endoscope model (4). The unique image properties further affect AI performance. Several previously developed AIs have been studied using images from a limited number of endoscope models (5–7). If the nature of the dataset used to validate the performance of the AI differs from that of the training dataset, it may cause errors (8). Therefore, outstanding performance may be degraded in images from other endoscope models. To develop practical AIs that can be applied generally, whether the image characteristics of various endoscope models that are not trained for AI affect performance should be investigated. Furthermore, the technical methods should be evaluated to overcome the identified performance degradation.

Deep learning-based domain adaptation algorithms, including cycle-consistent adversarial networks (CycleGAN), can interconvert the different image characteristics (9). This can be used to transform images into AI-trained styles and improve detection performance. The esophagogastric junction (EGJ) is a recommended site to be pictured during esophagogastroduodenoscopy (EGD) in clinical guidelines and is important for the diagnosis of reflux esophagitis or Barrett's esophagus (10, 11). In addition, the imaging characteristics of the squamous epithelium of the esophagus and columnar epithelium of the stomach appear together in pictures of the EGJ, which is crucial for AI development.

In the present study, we constructed EGD datasets for three different endoscope models. We checked whether AI can distinguish the models and investigated whether the image characteristics of the models influenced the EGJ detection performance of AI. Additionally, we determined whether this could be corrected using a domain adaptation algorithm.

## 2. Materials and methods

### 2.1. Collecting endoscopic images

We retrospectively collected cases of EGD performed between November 2015 and December 2020 at Soonchunhyang University Hospital, Seoul. The procedures were pictured using three endoscopic video processors: Olympus CV-260SL, CV-290 (Olympus, Tokyo, Japan), and PENTAX EPK-i (PENTAX Medical, Tokyo, Japan), each equipped with an exclusive endoscope. The other hardware and software involved in the image capture and storage of these examinations were identical and not involved in the image characteristics. The images were captured using a Matrox VIO 7 IA OA/G capture card (Matrox, Quebec, Canada) by duplication of the digital high-definition monitor output (1,920 × 1,080 pixels) from the video processors and then stored in a digital imaging and communication in medicine (DICOM)-compatible format in a picture archiving and communication system (PACS).

**Figure 1:**
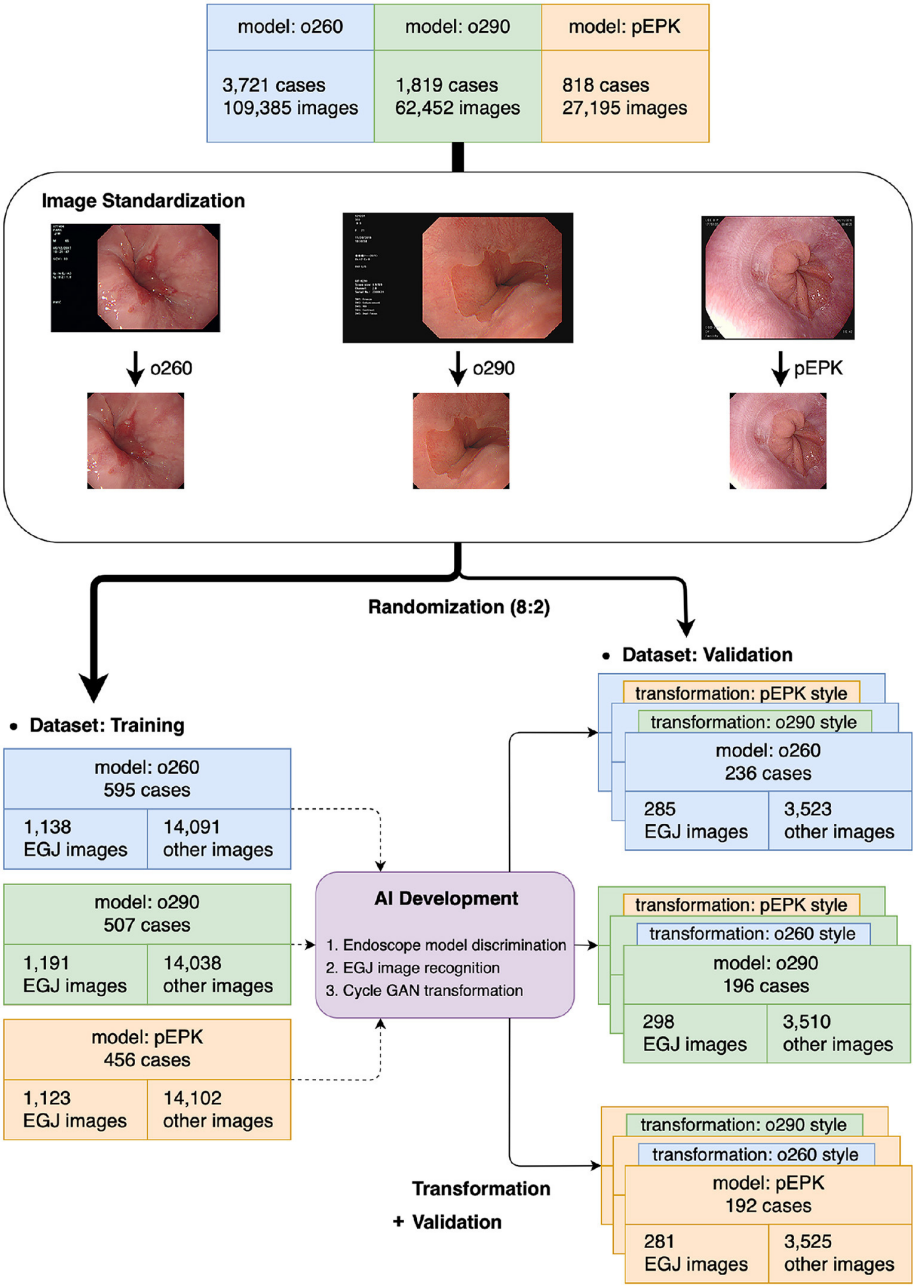
Flowchart of the present study. The images extracted from the original were distributed to the training and validation datasets at a ratio of 8:2 after the standardization process was completed. The AIs were trained using the training dataset and verified in the validation dataset and its transformed images using cycleGAN to obtain the characteristics of other endoscope models. o260, Olympus CV-260SL; o290, Olympus CV-290; pEPK, PENTAX EPK-i; AI, artificial intelligence; EGJ, esophagogastric junction; GAN, generative adversarial network.

The images stored in the PACS were extracted in Portable Network Graphics format, which supports full-color lossless data compression for AI training. The small border of the endoscopic field was cropped, whereas the images' subjects were retained. Finally, the images were standardized as octagonal images with a size of 512 × 512 pixels without losing their inherent characteristics, e.g., color, sharpness, and proportion (Figure 1). All images were anonymized, and the subsequent analysis protocols were approved by the local ethics committee of the Institutional Review Board (IRB, Soonchunhyang University Hospital, Seoul; No. 2020-05-010).

### 2.2. Experimental setting

Three nationally certified gastrointestinal endoscopy experts reviewed all cases and classified all images according to the video processor of the endoscope model in which they were captured. For accurate image classification, they referenced the part of the image containing the endoscope model information cropped during the standardization process. The images were labeled as o260, o290, and pEPK for Olympus CV-260SL, Olympus CV-290, and PENTAX EPK-i, respectively. Images whose characteristics were artificially modified using the image enhancement function, such as Olympus narrow band imaging (NBI) or PENTAX i-scan, were excluded from the study. Among the images of the lower esophagus, those expressing the Z-line of the epithelial squamocolumnar junction were labeled as EGJ images based on mutual agreement of the endoscopists. Moreover, the findings of reflux esophagitis including the Los Angeles classification and varices that can be visually confirmed on EGJ images were recorded by the endoscopists.

**Figure 2:**
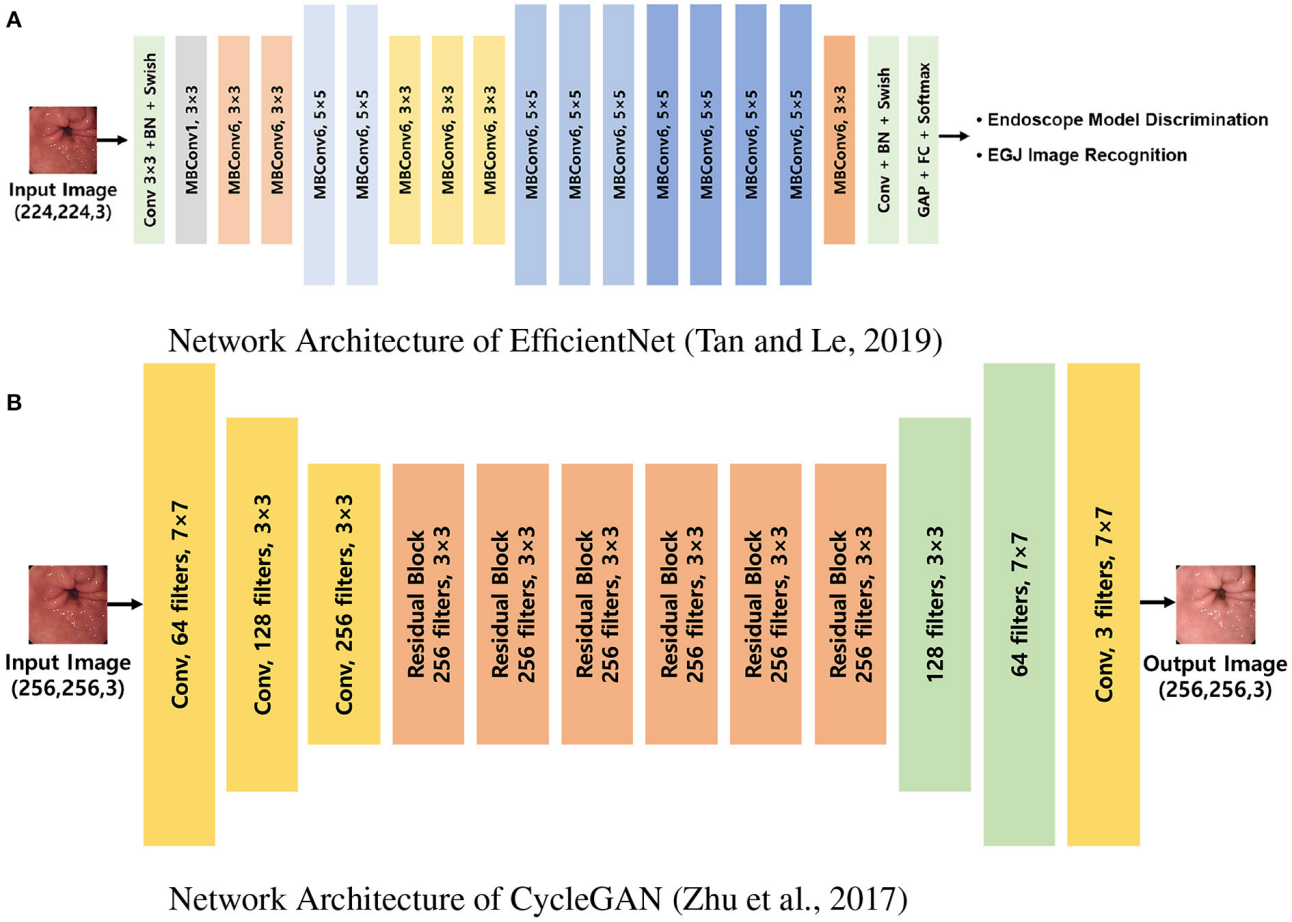
Network architectures used in the experiments. **(A)** Network architecture of EfficientNet (12). EfficientNet was trained to discriminate endoscope models and classify EGJ images for each model separately. **(B)** Network architecture of CycleGAN (9). CycleGAN was trained to transform images from a specific endoscope model to the others. MBConv, inverted linear BottleNeck layer with depth-wise separable convolution; BN, batch normalization; FC, fully connected layer; Conv, convolution; GAP, global average pooling; EGJ, esophagogastric junction; GAN, generative adversarial network.

After the standardization process, the images were randomly extracted by case to include a similar number of EGJ images when classified by each endoscope model. The images were distributed in an approximately 8:2 ratio such that no cases intersected with one another and were classified into training and validation datasets, respectively. Two types of AI were developed to distinguish the endoscope model used for imaging and determine whether they were EGJ images using the training dataset. An AI that discriminates the endoscope models was trained using the entire training dataset; it labeled the images into three classes according to the endoscope models. Another AI to detect EGJ images was independently optimized for the three different endoscope models by training a separate dataset for each endoscope model. All AIs were based on the EfficientNet-B0 model (Figure 2A), which has been proven to be efficient and accurate (12). The pre-trained model using ImageNet was incorporated as an initial parameter. The size of an input image for EfficientNet was 224 × 224 pixels. Stochastic gradient descent was used for training with 0.05 learning rate, 0.9 of momentum, and 1*e*^−4^ of weight decay. Cross-entropy loss was used to solve the classification problem. The best model to show the highest accuracy for the validation dataset was selected as the final model during 200 epochs.

Computational processes were implemented using a workstation with NVIDIA RTX2080 (NVIDIA, CA, USA) cards and 8-GB memory. The image characteristics of each endoscope model for the cycleGAN transformation were instructed using the same training dataset (Figure 2B), and the images of the validation dataset were restyled to have the characteristics of the two models that differed from those of the original (9). The size of an input image for cycleGAN was 256 × 256 pixels. Adam optimizer was used for training with a 0.0002 learning rate. Given there is no ground truth for evaluating the performance of image transformation, the final model was determined after training of 200 epochs. The endoscopists reviewed all the images to ensure that the EGJ images were correctly identified, even in the converted images.

The AI distinguished the endoscope model, in which a picture was validated with the highest softmax value for top-1 accuracy. Another AI representing the probabilities of EGJ images was validated with a binary classification threshold of 0.5 in both the validation set and those of cycleGAN-transformed images. To qualitatively evaluate the mechanism of action of EGJ recognition by the AI, a class activation map was created on the regions corresponding to the EGJ and endoscopy experts confirmed that it was recognized as intended (13).

### 2.3. Statistical analysis

Artificial intelligence performances were evaluated numerically based on accuracy and F1-scores and calculated using SPSS (IBM SPSS Statistics for Windows, version 26.0; IBM Corp., Armonk, NY, USA) software. They were compared statistically through receiver operating characteristic (ROC) curve comparisons of DeLong's test using MedCalc software (MedCalc, version 20.100; MedCalc Software Ltd., Ostend, Belgium) (14). Statistical significance was set at *P* < 0.05.

## 3. Results

The results of 6,358 examinations in total were collected, and 2,160 cases were randomized for AI development and validation (Table 1). The mean age of patients at the time of endoscopy was 54.7 years; 970 patients were women. Furthermore, 831 cases were examined using Olympus CV-260SL and comprised 19,037 images, of which 1,423 were EGJ images. Additionally, 681 cases from Olympus CV-290 yielded 19,037 images, including 1,489 EGJ images. In 648 PENTAX EPK-i cases, 1,404 of the 19,031 images were labeled as EGJ images. Among the selected cases, 605 patients had reflux esophagitis and 14 had esophageal varices.

**Table 1:** Clinical characteristics of randomly distributed case.

**Case** **(Image)**	**Endoscope** **model**	**o260**		**o290**		**pEPK**		
	**Dataset**	**Training**	**Validation**	**Training**	**Validation**	**Training**	**Validation**
Total		595 (15,229)	236 (3,808)	507 (15,229)	174 (3,808)	456 (15,225)	192 (3,806)
Sex						
Male		333 (8,633)	132 (2,125)	275 (8,108)	131 (2,893)	215 (7,364)	104 (2,118)
Female		262 (6,596)	104 (1,683)	232 (7,121)	43 (915)	241 (7,861)	88 (1,688)
Age at endoscopy (years, mean±SD)		49.6 ± 12.5	49.8 ± 12.2	58.7 ± 14.4	56.0 ± 13.2	57.7 ± 12.1	57.9 ± 12.5
Clinical feature of EGJ							
Normal		491 (895)	190 (218)	392 (851)	8 (10)	335 (756)	139 (193)
Reflux esophagitis							
LA-A		75 (168)	39 (57)	85 (241)	88 (143)	94 (256)	41 (63)
LA-B		24 (65)	7 (10)	27 (78)	62 (115)	21 (87)	10 (24)
LA-C		4 (8)	0 (0)	3 (21)	10 (21)	4 (14)	2 (1)
LA-D		1 (2)	0 (0)	0 (0)	6 (9)	2 (10)	0 (0)
Esophageal varix		2 (5)	0 (0)	3 (10)	3 (6)	4 (11)	2 (4)

o260, Olympus CV-260SL; o290, Olympus CV-290; pEPK, PENTAX EPK-i; EGJ, esophagogastric junction; SD, standard deviation; LA, Los Angeles classification.

**Figure 3:**
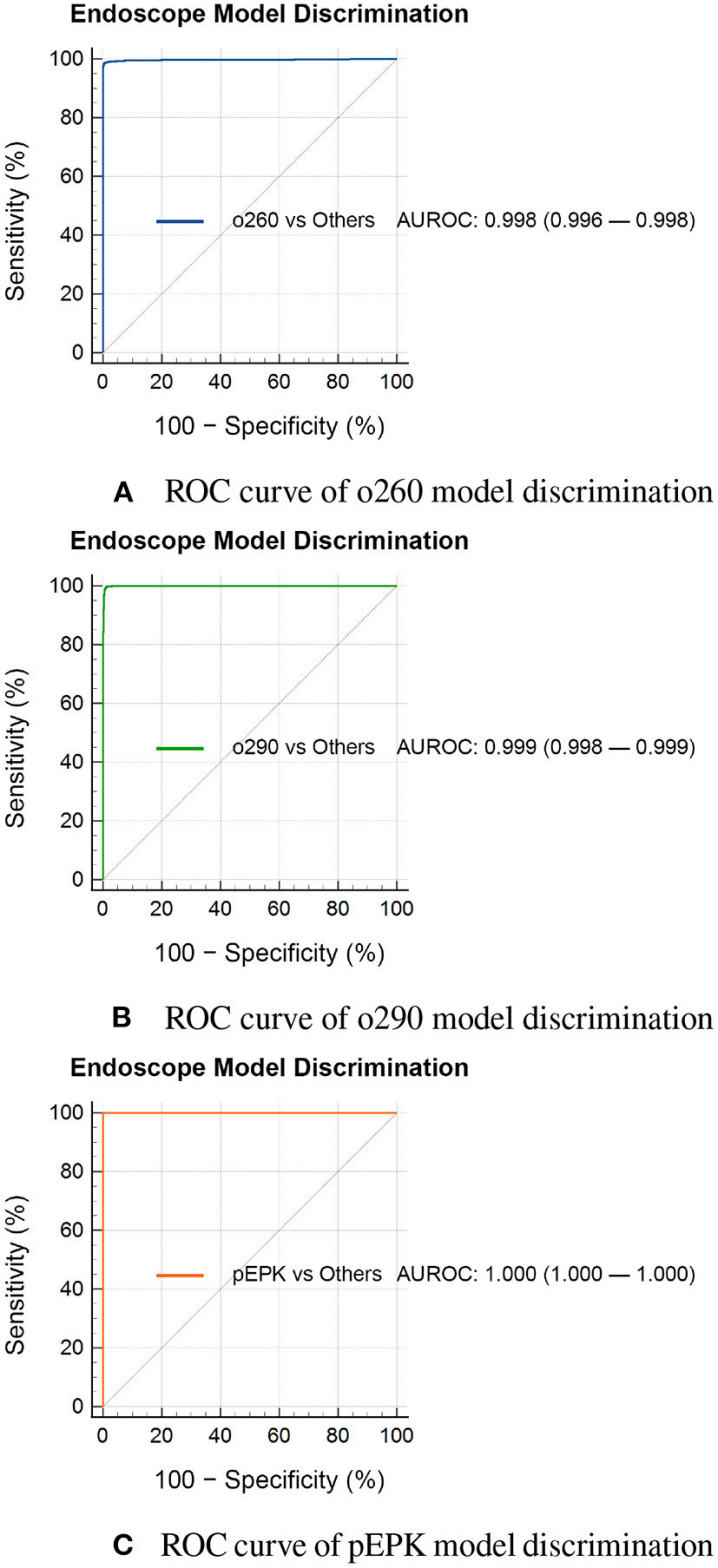
Performance of endoscope model discrimination AI. ROC curve of **(A)** o260, **(B)** o290, and **(C)** pEPK model discrimination. The AI predicting the endoscope model that captured the images, had errors in determining **(A)** 93 images in the o260 dataset set were pictured with an Olympus CV-290 and **(B)** 14 images in the o290 dataset were taken with an Olympus CV-260SL. **(C)** It successfully determined all the pEPK dataset images captured with PENTAX EPK-i. o260, Olympus CV-260SL; o290, Olympus CV-290; pEPK, PENTAX EPK-i; AUROC, area under the receiver operating characteristic curve.

Artificial intelligence for endoscope model discrimination distinguished the PENTAX EPK-i model images from those of the Olympus model images without faults. The AI showed errors in predicting 93 images of the o290 dataset as those of the o260 dataset and 14 of the o260 as o290. The top-1 accuracy of the AI was 0.991, and the F1-scores for the o260, o290, and pEPK values were 0.986, 0.986, and 1.000, respectively. The areas under receiver operating characteristic curves (AUROCs) for model prediction were 0.998, 0.999, and 1.000 for the o260, o290, and pEPK models, respectively (Figure 3).

**Figure 4:**
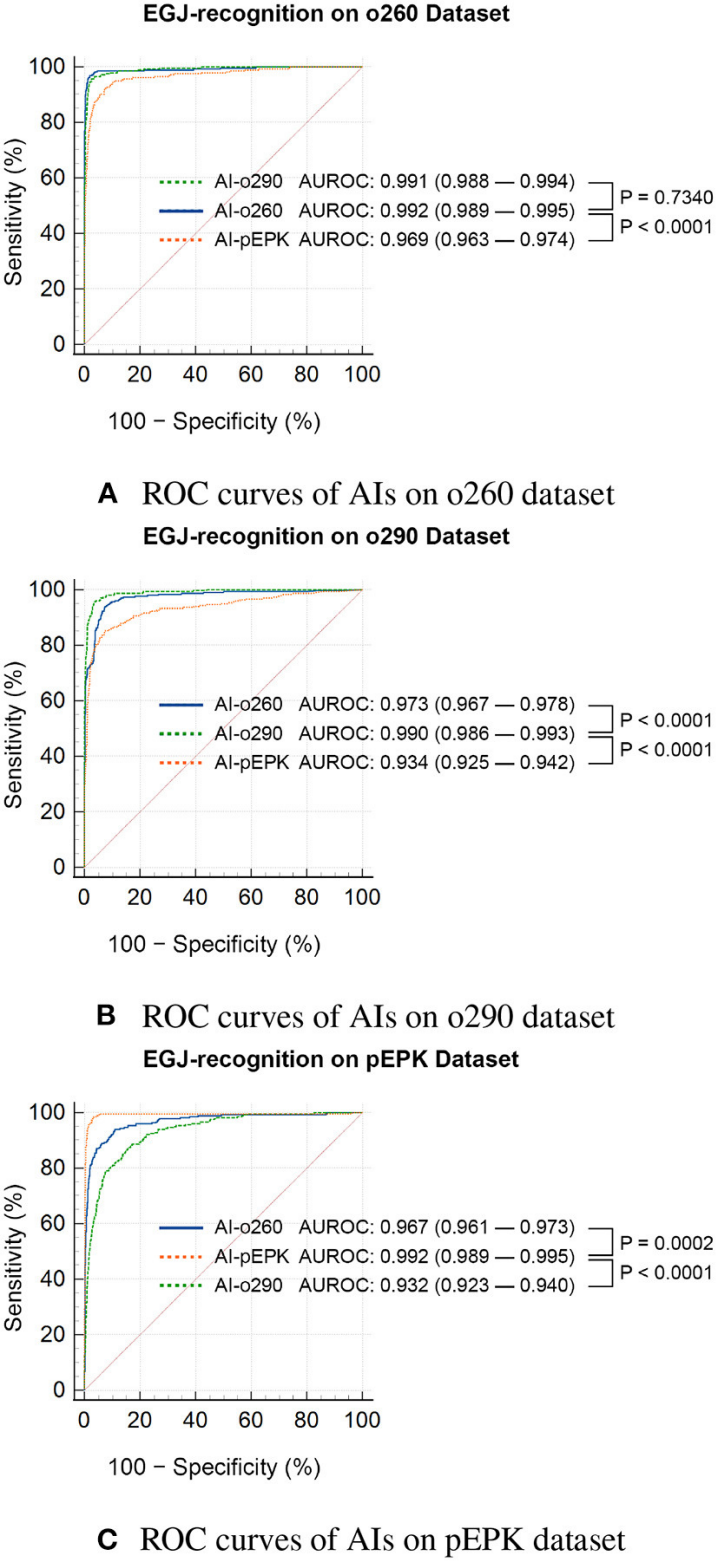
Statistical comparison of the ROC curves of EGJ-recognition AIs on validation datasets. ROC curves of AIs on **(A)** o260, **(B)** o290, and **(C)** pEPK dataset. EGJ-recognition AIs (AI-o260, AI-o290, and AI-pEPK) trained on a dataset classified by the endoscope model had degraded performance on the validation dataset of other endoscope models. **(A)** The performance difference between AI-o260 and AI-o290 in this dataset was the only nonsignificant result. **(B,C)** In the o290 and pEPK dataset, the performance of AIs trained with images of different endoscope models were significantly inferior. o260, Olympus CV-260SL; o290, Olympus CV-290; pEPK, PENTAX EPK-i; AI, artificial intelligence; EGJ, esophagogastric junction; AUROC, area under the receiver operating characteristic curve.

Three AIs that recognized the EGJ were created and named AI-o260, AI-o290, and AI-pEPK after the optimized endoscope models. All AIs exhibited the highest accuracy and F1-score for the images of the validation set pictured with the same endoscope model as that used to obtain the trained images. The AI-o260 recognized 285 EGJ images of the o260 validation set with an accuracy of 0.988, and the F1-score was 0.917 (Table 2). The AI-o290 exhibited an accuracy of 0.979 in identifying the images of the o290 set, and the F1-score was 0.877. The accuracy of AI-pEPK for the validation set of pEPK and F1-score were 0.986 and 0.906, respectively. The AIs exhibited worse performance on other validation datasets that were pictured with an endoscope model different from that of the training set (Figure 4). Comparing the AUROC of each AI, all differences were statistically significant, except for the results of AI-o260 and AI-o290 on the o260 dataset.

**Table 2:** Comparison of EGJ-recognition performance by AIs in classified validation datasets.

	**o260 Dataset**		**o290 Dataset**		**pEPK Dataset**	
	**Total** **image** **count**	**EGJ** **image count**	**Total** **image** **count**	**EGJ** **image count**	**Total** **image** **count**	**EGJ** **image count**
	3,523	285	3,510	298	3,525	281
	**Original** **images**		**Original** **images**		**Original** **images**	
**AI**	**Accuracy**	**F1-score**	**Accuracy**	**F1-score**	**Accuracy**	**F1-score**
AI-o260	0.988	0.917	0.950	0.697	0.961	0.757
AI-o290	0.981	0.877	0.979	0.867	0.936	0.596
AI-pEPK	0.963	0.703	0.945	0.527	0.986	0.906
	N/A	**Transformed images** **like o260**		**Transformed images** **like o260**	
		**Accuracy**	**F1-score**	**Accuracy**	**F1-score**
AI-o260	–	0.975	0.839	0.986	0.902
	**Transformed images** **like o290**		N/A	**Transformed images** **like o290**	
	**Accuracy**	**F1-score**		**Accuracy**	**F1-score**
AI-o290	0.988	0.914	–	0.986	0.902
	**Transformed images** **like pEPK**		**Transformed images** **like pEPK**		N/A
	**Accuracy**	**F1-score**	**Accuracy**	**F1-score**	
AI-pEPK	0.987	0.906	0.973	0.805	–

o260, Olympus CV-260SL; o290, Olympus CV-290; pEPK, PENTAX EPK-i; AI, artificial intelligence; EGJ, esophagogastric junction.

The AIs (AI-o260, AI-o290, and AI-pEPK) were named after the endoscope model that captured the training dataset.

**Figure 5:**
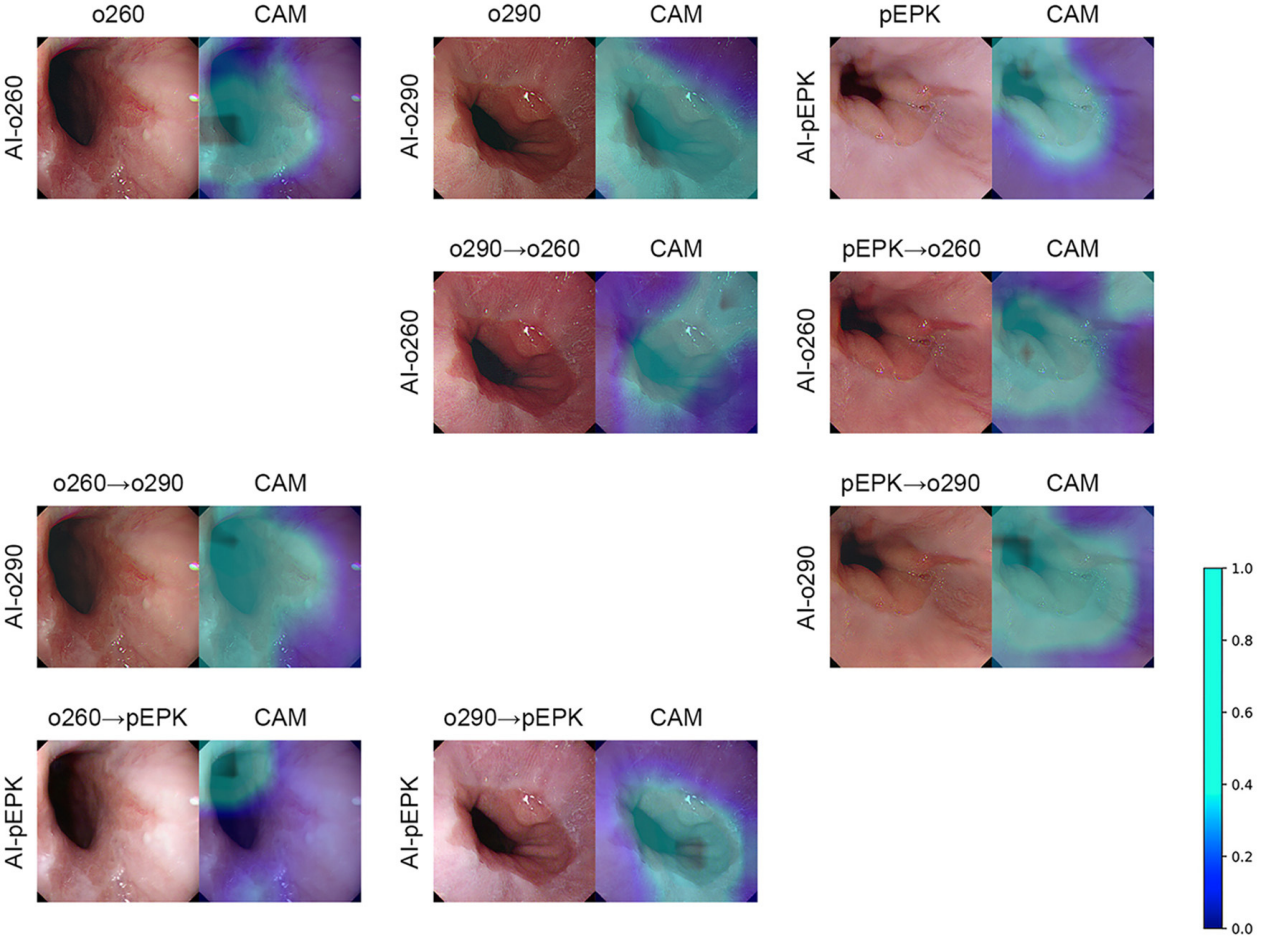
Examples of CycleGAN transformation and class activation map of EGJ-recognition AI. The AI indicated on the far-left side of the figure generates the CAM based on the endoscopic image placed on the left. As shown in the color scale bar on the right, a light blue color on the CAM denotes a higher significance level. The overlaying result is arranged on the right side of the figure. The figures in the top row are the results of the original endoscope model images. The cycleGAN transformation results are listed in the following three rows. The transformation was performed on the top original images to obtain the image characteristics of the AI indicated on the far-left side of the figure. o260, Olympus CV-260SL; o290, Olympus CV-290; pEPK, PENTAX EPK-i; AI, artificial intelligence; EGJ, esophagogastric junction; CAM, class activation map; GAN, generative adversarial network.

**Figure 6:**
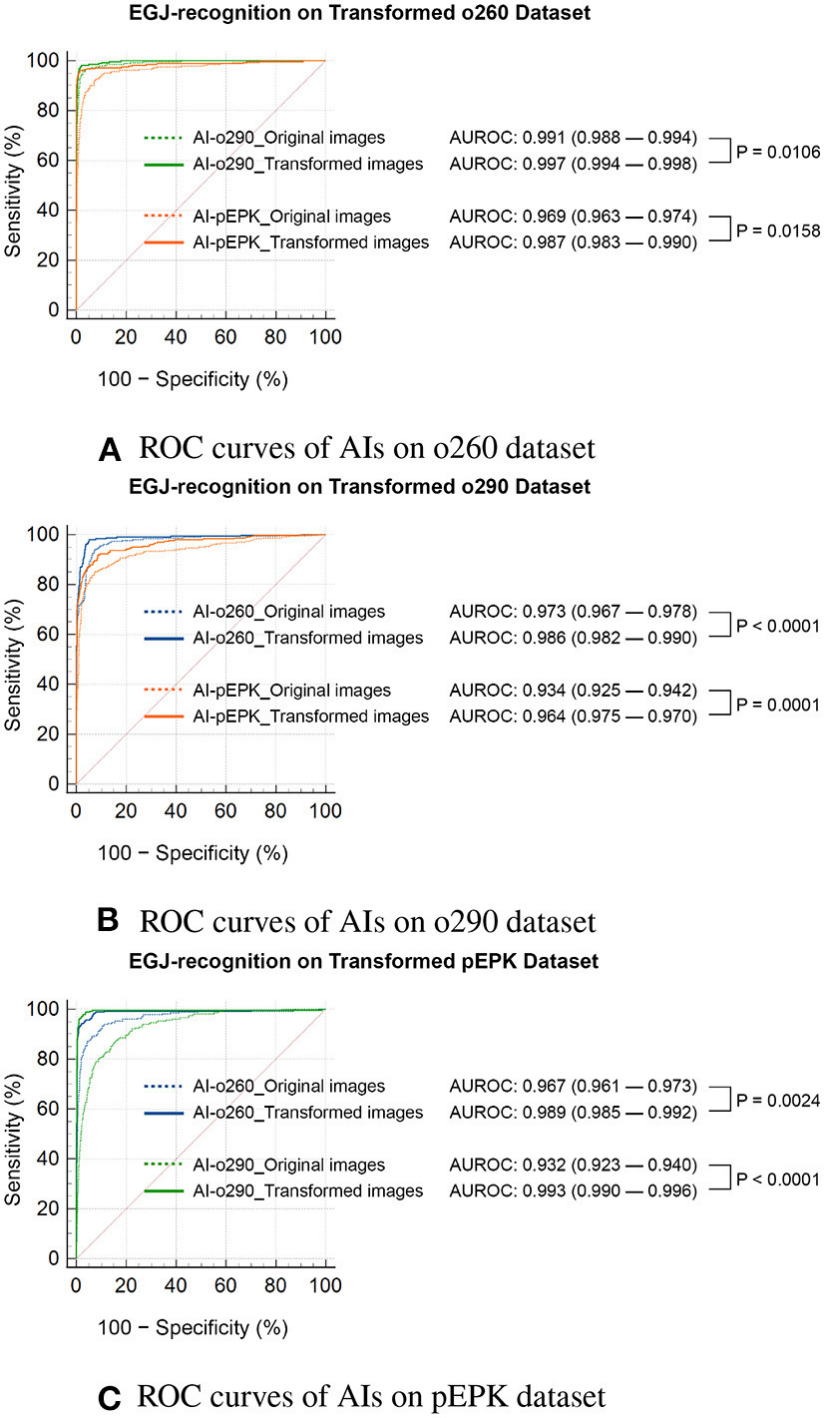
Statistical comparison of the ROC curves of EGJ-recognition AIs on transformed validation datasets. ROC curves of AIs on **(A)** o260, **(B)** o290, and **(C)** pEPK dataset. Compared with the results on the original dataset (indicated by dotted lines), the EGJ-recognition AIs performed better in the transformed dataset (indicated by solid lines) to represent the image characteristics used in the training process. **(A,B)** The recognition rate of AIs improved on the transformed Olympus endoscope images to optimize for the model the AI trained on. The AI trained with PENTAX endoscope images showed greater improvement. **(C)** The performance of AIs trained with Olympus endoscope images also improved on the transformed PENTAX endoscope images. o260, Olympus CV-260SL; o290, Olympus CV-290; pEPK, PENTAX EPK-i; AI, artificial intelligence; EGJ, esophagogastric junction; AUROC, area under the receiver operating characteristic curve.

There was no change in the labeling of the EGJ images after transforming the original three datasets to have the characteristics of the other two endoscope models. Esophagogastric junction-recognition AIs were presumed to recognize EGJ by identifying the epithelial squamocolumnar junction's boundary line (Figure 5). Esophagogastric junction recognition AIs exhibited higher AUROC values in the transformed images, similar to the characteristics of their trained images (Table 2). All improvements were statistically significant (Figure 6). AI-o290 showed an AUROC improvement of 0.0056 (*P* = 0.0106) in the converted o260 dataset with o290 characteristics, and AI-pEPK exhibited an improvement of 0.0182 (*P* = 0.0158) in the converted o260 dataset, such as pEPK images. Compared with the values in the original images of the o290 dataset, the AUROC values of AI-o260 and AI-pEPK were 0.0134 (*P* < 0.0001) and 0.0299 (*P* = 0.0001) in the transformed images with the characteristics of o260 or pEPK, respectively. When the pEPK dataset was changed to fit the o260 and o290 characteristics, the improved AUROC values for AI-o260 and AI-o290 were 0.0215 (*P* = 0.0024) and 0.0616 (*P* < 0.0001), respectively.

## 4. Discussion

Endoscopic examinations are recorded using photographs of specific compartments in accordance with the recommended guidelines and additional detailed observations of the detected lesions. Images are captured using several established devices. Everything regarding the hardware, including light sources, lenses, and sensors, to the software that processes and stores signals, relates to the style of the endoscopic images, which is also the basis of the manufacturers' unique technology. These differences create exclusive image characteristics for each endoscope model. All endoscope models used in this study have an observation field of view of 140°, use a white Xenon lamp as a light source, and have a maximum field depth of 100 mm, so the optical characteristics are similar (15, 16). Compared to the PENTAX EPK-i, which directly senses a white light, Olympus models express white by recombination of three color lights collected through physical filters, resulting in a distinctive color difference (17). In addition, the image sensors of the endoscope models, which are divided into complementary metal-oxide semiconductor or charge-coupled device, result in differences in image resolution and noise aspects (18). Moreover, the exclusive software functions that process the signals also contribute to differences in the images. These can be easily distinguished by experienced endoscopists.

The endoscopic images in the current study were all preprocessed into images of the same size, leaving only the inspection area. Even in the standardized images, the differences can be distinguished by experienced endoscopists and AI. Artificial intelligence only made 107 errors among the 7,616 images obtained using the Olympus models. In cases where more distinct differences in image characteristics were present due to different endoscope manufacturers, AI distinguished these differences without error. The calculated performances were sufficiently high with F1 scores of 0.98 or higher and AUROCs of 0.99 or higher for all datasets of the three endoscope models. These results can be interpreted as the existence of distinctive image characteristics, and the difference is particularly evident when the manufacturers differ.

Deep learning-based AI makes inductive decisions based on a large amount of data. Developers can customize the functions of AI in the way they expect by labeling the training materials. However, if the data contain classifiable characteristics independent of labels, unintended consequences can occur. The image characteristics of the endoscope models that AI inadvertently learns may influence performance. Zippelius et al. have investigated the GI Genius (Medtronic, Minneapolis, MN, USA) system in a comparative study of colonoscopy using the Olympus 190 model and acquired a result not inferior to an adenoma detection rate (ADR) of 50.7% (7). In contrast, Repici et al. used images that were captured with two models, Olympus 190 and Fujifilm ELUXEO 700 (FUJIFILM, Tokyo, Japan), to report the effectiveness of the same GI Genius in a similar randomized trial (19); a higher ADR (54.8%) was obtained with AI assistance than that in the study's control group. We do not know what dataset the GI Genius is based on or how the ratio of the two types of endoscopic images was used in Repici et al.'s study; however, we know that different conclusions can be drawn from the different nature of the data.

Although most clinical studies on lesion detection in endoscopic images have been conducted using the open-source AI algorithm, the excellent results are highly likely to be obtained only by a few Japanese companies that oliogopolize the gastrointestinal endoscopy market. Ruan et al. have reported a high identification accuracy of a deep learning diagnostic system for inflammatory bowel disease (20). All endoscopic examinations were performed using an Olympus CV-290SL model. Ebigbo et al. have predicted the submucosal invasion of Barrett's cancer using AI, based on endoscopic images obtained using the Olympus 190 model (21). Considering the results of our research, AIs may show poorer performance on images of other endoscope models.

In the field of image recognition research using DL, performance improvement through algorithm remodeling has reached a plateau, and the significance of organizing the data used in the training and validating process of DL has emerged. Ng et al. introduced a data-centric AI campaign to overcome the robustness of deep neural networks (22). The task force of the American Society of Gastrointestinal Endoscopy has raised the need for a standardized database named Endonet, which is managed by experts (23).

In the present study, we not only identified the performance problem of AI that learned biased data but also presented a solution using a different AI. If endoscopic imaging is compared to a work of art, the endoscope model is a painter, and the imaging characteristic is the style of painting. Developing a conversion algorithm of painting style through quantitative analysis is difficult. However, AI can inductively find a method of transformation. Zhu et al. have successfully converted photographs to appear like paintings by Monet or Van Gogh using a DL-based domain adaptation technology called cycleGAN (9). We used the algorithm to convert datasets to obtain the image characteristics of endoscope models that were familiar to EGJ-recognition AI, resulting in improved performance.

The current study retrospectively analyzed data from a single institution. It can be pointed out that these aspects undermine the research's objectivity. However, these inevitable deficiencies made strict data management possible. This study was conducted in a rare endoscopy center that utilizes various endoscopes from various manufacturers. The process of capturing endoscopic signals to store them in the standard DICOM format was consistently managed using the same instruments. Moreover, the datasets for training and validation were completely separated such that there was no correlation at a level comparable to that of external datasets. Extracted endoscopic images were cropped to the same size and shape so that only the intrinsic image characteristics of the endoscope models could affect AI performance. Errors could be reduced by the selection of an object that was distinguishable because of its histological characteristics as a feature to be detected and determining them with the consensus of multiple observers. It has successfully achieved a high detection rate based on strictly managed data, although the AI used in the current study is a lightweight model that uses relatively few parameters. In order to empower the hypothesis of this study, we presented similar results from experiments using separately randomized datasets as Supplementary Materials. There were also differences in the images that AI could distinguish depending on the endoscope models in other datasets. It was confirmed that the recognition performance of AI could be affected by the different image characteristics of different endoscope models.

It can be claimed that limiting only the EGJ image as a recognition target in this study reduces the clinical significance of our results. However, it is an excellent feature to validate the detection performance of AI. The EGJ contains relevant clinical information and can be easily identified by the clear boundary. Recognizing the EGJ itself is just as significant as directly identifying the various diseases that exist in the region. Although it is not a subject of analysis in this study, it can be seen that our dataset consists of a realistic composition through the disease incidences that can be visually confirmed at the site. Artificial intelligence that finds lesions in endoscopic images must be accustomed to filtering out images unrelated to the recognition target that occupy larger numbers. The EGJ image, which the clinical guidelines recommend to be pictured, occupies a relatively small part of the total endoscopic images. We attempted to construct the dataset considering the quantitative imbalance of the detection target and correctly demonstrate the performance of AI using accuracy and F1-score. These attempts enable more realistic AI performance evaluation.

Once DL-based AI is fully developed, it is difficult to change the training dataset. Optimization of AI using image transformation should be applied to the dataset to be analyzed by AI. All AIs in this study were developed and validated on an identical dataset to ensure consistency with one another. The cycleGAN-based transformation was applied to the validation dataset, which was the target of AI analysis, and AUROC was used to facilitate accurate statistical comparisons of its performance. In particular, the performance of AI was prominently improved by transforming the image of other endoscope manufacturers from what the AI trained. The result that the performance improvement of binary classification AI can reach up to 6% with the cycleGAN transformation is also of practical value.

We used endoscope models to demonstrate that distinguishable image properties existed. Although these details may seem insignificant, the classifiable image characteristics of the dataset that are not addressed in the training stage may affect AI performance. In the present study, the AI was trained to be biased toward images of a specific endoscope model and showed a decreased detection rate in images of other endoscope models. Further studies to find these obscure details should be supported to develop more practical AI. Furthermore, we significantly improved AI performance by converting images into a style familiar to the AI using a domain adaptation technology called cycleGAN. A domain is all the values that can go into a function with its given contexts; in the field of image recognition using DL, it refers to classifiable features concealed in images. Cycle-consistent adversarial network discriminates domains without detailed instructions from researchers based on large amounts of data (24). Moreover, cycleGAN can convert the trained domains to have the characteristics of other domains. Even though the accuracy of DL-based image recognition has gradually increased, the handling of different domain data remains a challenging task. In this study, we showed that a domain adaptation method like cycleGAN can reduce the performance gap when the DL model is applied to other domains. To develop a universal DL method that can be applicable for most of endoscope models, image recognition and domain adaptation should be dealt with simultaneously.

## Data availability statement

The datasets presented in this article are not readily available because the images can be reconstructed and compromise privacy. Requests to access the datasets should be directed to JP, junspark@schmc.ac.kr.

## Ethics statement

All images used in this study were anonymized, and the subsequent analysis protocols were approved by the local Ethics Committee of the Institutional Review Board (IRB, Soonchunhyang University Hospital, Seoul; No. 2020-05-010). Written informed consent from the (patients/participants OR patients/participants legal guardian/next of kin) was not required to participate in this study in accordance with the national legislation and the institutional requirements.

## Author contributions

JP, YH, and HK contributed to conception and design of this study. YH, SK, JP, HK, JL, J-OK, TL, SJ, SH, and BK helped with data collection, analysis, and interpretation. This article was drafted by JP and critical revisions of critical points were made by YH. HK finally approved this article. All authors contributed to the article and approved the submitted version.

## Funding

This work was supported by the National Research Foundation of Korea (NRF) grant funded by the Korean Government (MSIT) (no. 2020R1G1A1009221) and Soonchunhyang University Research Fund (no. 2022-1031).

## Conflict of interest

The authors declare that the research was conducted in the absence of any commercial or financial relationships that could be construed as a potential conflict of interest.

## Publisher's note

All claims expressed in this article are solely those of the authors and do not necessarily represent those of their affiliated organizations, or those of the publisher, the editors and the reviewers. Any product that may be evaluated in this article, or claim that may be made by its manufacturer, is not guaranteed or endorsed by the publisher.
