# Supplementary material for: Reduced detection rate of artificial intelligence in images obtained from untrained endoscope models and improvement using domain adaptation algorithm
Source: Front Med (Lausanne). 2022 Nov 10;9:1036974. doi: 10.3389/fmed.2022.1036974 (PMC9684642; doi:10.3389/fmed.2022.1036974)
Supplement: Supplementary file 1 [file Data_Sheet_1.PDF]

Supplementary Material

1 SUPPLEMENTARY DATA

To demonstrate the objectivity of the study, five additional experiments were performed on a completely new randomized dataset. The AIs, which discriminates the endoscope model used for imaging, showed excellent model recognition levels, exceeding AUC 0.9 on 15 occasions (Fig 1, 2, 3, 4, 5). In the each of 5 experiments, 3 datasets were generated according to the model of endoscope, and a total of 45 EGJ image-recognition experiments were performed on AIs trained in a biased manner. All AIs showed the highest EGJ recognition performance in images pictured with the same model of endoscope they were trained with (Table S1). Fifteen experiments comparing the AI performances for each dataset was conducted, and statistical significance was confirmed in 7 of them (Fig 6, 7, 8, 9, 10). Differences in AI recognition performance showed a consistent tendency to support the hypothesis that such differences may be influenced by image characteristics according to the endoscope model.

2 SUPPLEMENTARY TABLES AND FIGURES

2.1 Table

Table S1. Comparison of Experimental Values of Other Randomized Dataset

		Endoscope Model	o260		o290		pEPK	
		**Dataset**	**Training**	**Validation**	**Training**	**Validation**	**Training**	**Validation**
**Experiment 01**		**Total Case**	2,920	733	1,745	433	535	134
		**EGJ Image**	1,126	224	1,128	242	1,125	256
		**Total Image**	17,442	4,548	17,422	4,630	17,418	4,661
	**AI-o260**	Accuracy	0.968		0.948		0.946	
		F1-score	0.633		0.435		0.432	
	**AI-o290**	Accuracy	0.948		0.948		0.944	
		F1-score	0.544		0.552		0.503	
	**AI-pEPK**	Accuracy	0.945		0.941		0.976	
		F1-score	0.365		0.354		0.788	
**Experiment 02**		**Total Case**	2,930	732	1,744	434	534	134
		**EGJ Image**	1,119	265	1,124	244	1,118	264
		**Total Image**	17,779	4,484	17,781	4,434	17,779	4,464
	**AI-o260**	Accuracy	0.947		0.942		0.954	
		F1-score	0.564		0.386		0.577	
	**AI-o290**	Accuracy	0.948		0.956		0.945	
		F1-score	0.380		0.468		0.310	
	**AI-pEPK**	Accuracy	0.948		0.941		0.970	
		F1-score	0.587		0.473		0.758	
**Experiment 03**		**Total Case**	2,928	728	1,745	434	534	135
		**EGJ Image**	1,120	217	1,122	231	1,125	250
		**Total Image**	18,074	3,826	18,077	4,251	18,079	4,295
	**AI-o260**	Accuracy	0.965		0.940		0.938	
		F1-score	0.668		0.387		0.396	
	**AI-o290**	Accuracy	0.956		0.955		0.954	
		F1-score	0.487		0.462		0.372	
	**AI-pEPK**	Accuracy	0.941		0.942		0.964	
		F1-score	0.410		0.411		0.716	
**Experiment 04**		**Total Case**	2,920	733	1,740	434	535	134
		**EGJ Image**	1,060	227	1,055	198	1,060	277
		**Total Image**	17678	4101	17674	3793	17690	4105
	**AI-o260**	Accuracy	0.963		0.952		0.947	
		F1-score	0.653		0.425		0.409	
	**AI-o290**	Accuracy	0.948		0.972		0.944	
		F1-score	0.421		0.579		0.437	
	**AI-pEPK**	Accuracy	0.937		0.944		0.971	
		F1-score	0.422		0.385		0.788	
**Experiment 05**		**Total Case**	2,915	723	1,744	432	534	135
		**EGJ Image**	1016	248	1013	232	1014	276
		**Total Image**	17,552	3,527	17,555	3,604	17,557	3,593
	**AI-o260**	Accuracy	0.960		0.940		0.938	
		F1-score	0.643		0.405		0.481	
	**AI-o290**	Accuracy	0.946		0.955		0.935	
		F1-score	0.555		0.567		0.420	
	**AI-pEPK**	Accuracy	0.943		0.923		0.962	
		F1-score	0.549		0.337		0.759	

o260, Olympus CV-260SL; o290, Olympus CV-290; pEPK, PENTAX EPK-i; AI, artificial intelligence; EGJ, esophagogastric junction

The AIs (AI-o260, AI-o290, and AI-pEPK) were named after the endoscope model that captured the training dataset.

2.2 Figures

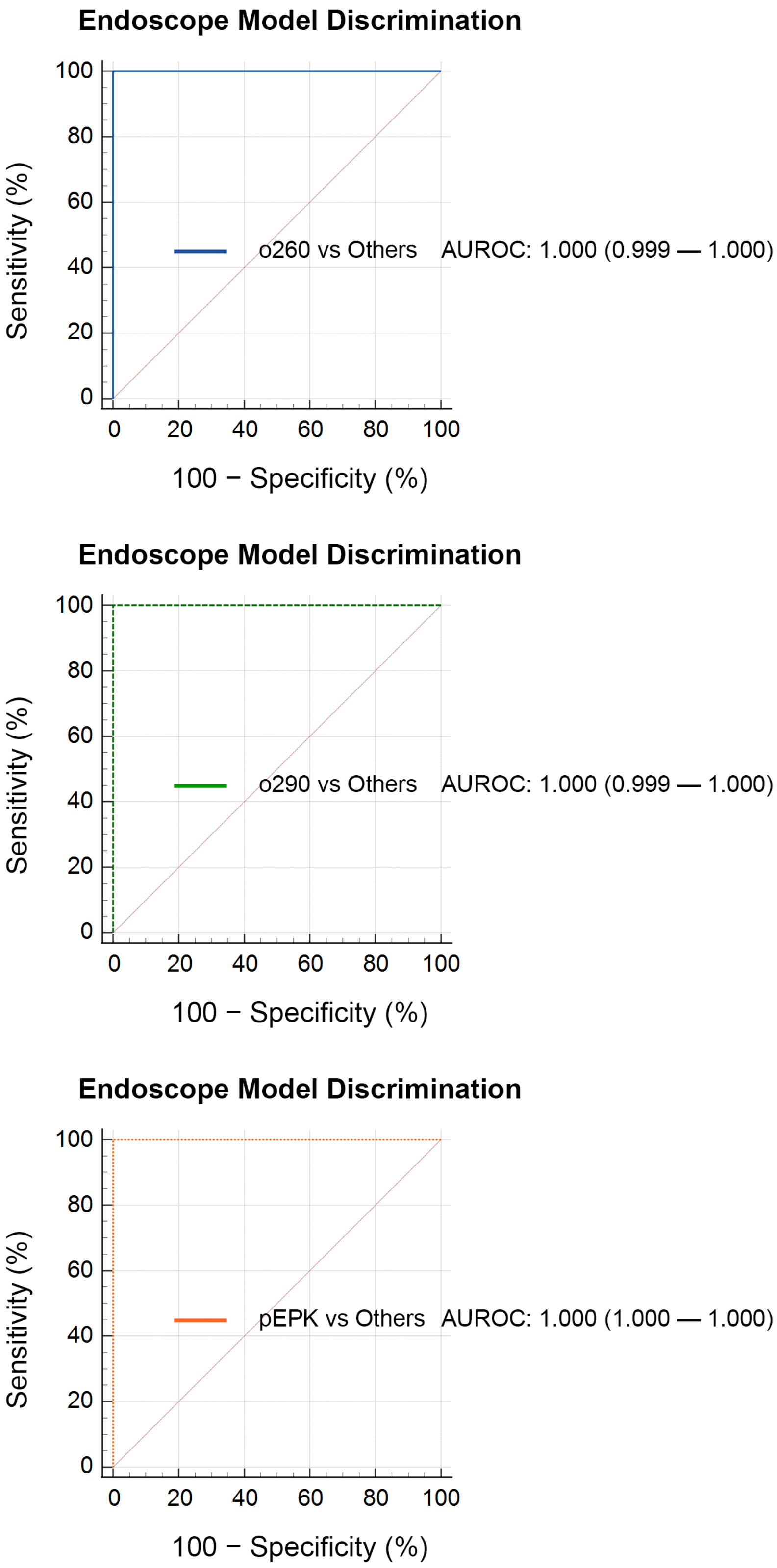

Figure S1. Performance of Endoscope Model Discrimination AI (Experiment 01). All AIs successfully determined the endoscope model that captured images.

o260, Olympus CV-260SL; o290, Olympus CV-290; pEPK, PENTAX EPK-i; AUROC, area under the receiver operating characteristic curve

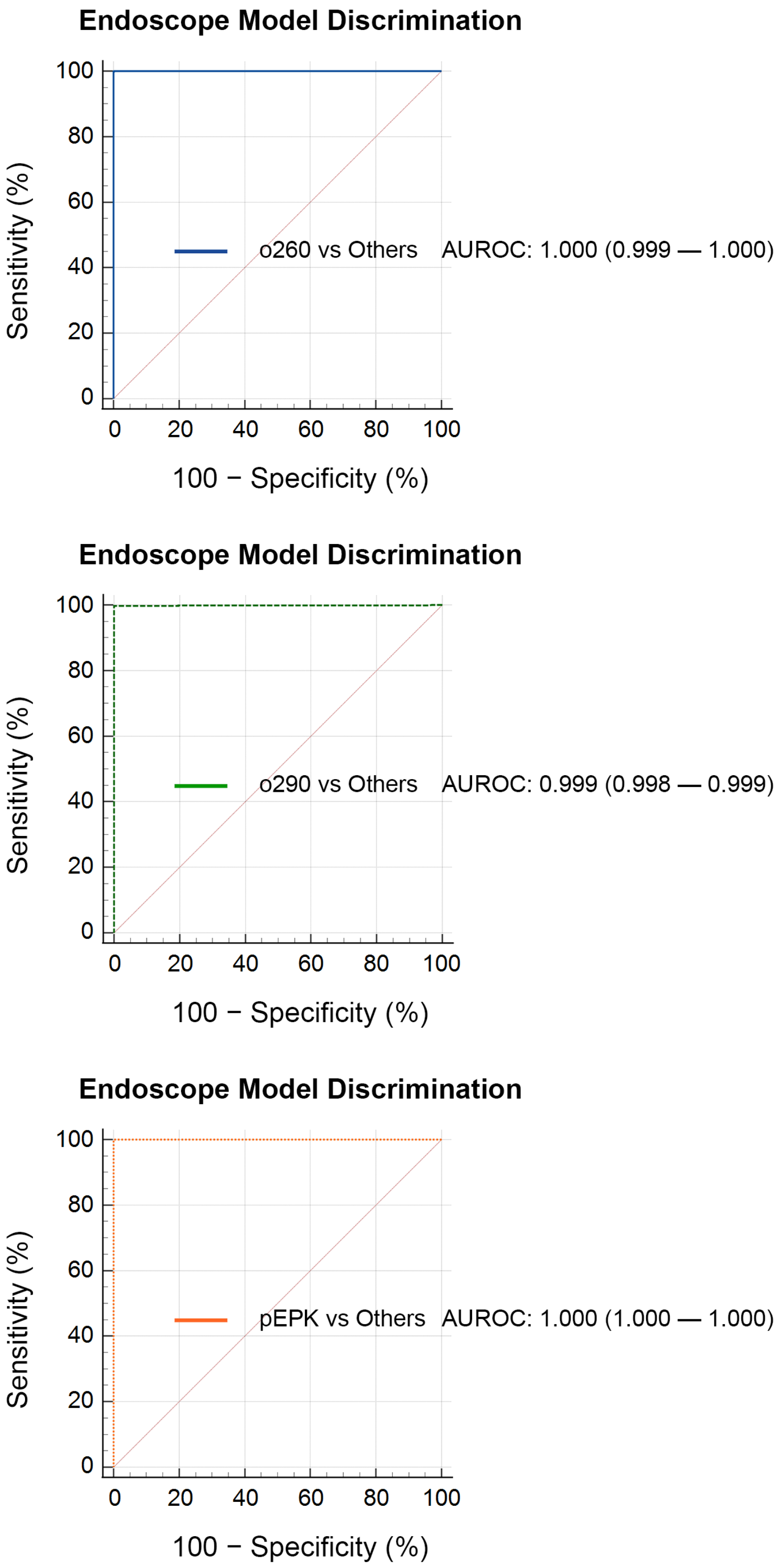

Figure S2. Performance of Endoscope Model Discrimination AI (Experiment 01). All AIs successfully determined the endoscope model that captured images.

o260, Olympus CV-260SL; o290, Olympus CV-290; pEPK, PENTAX EPK-i; AUROC, area under the receiver operating characteristic curve

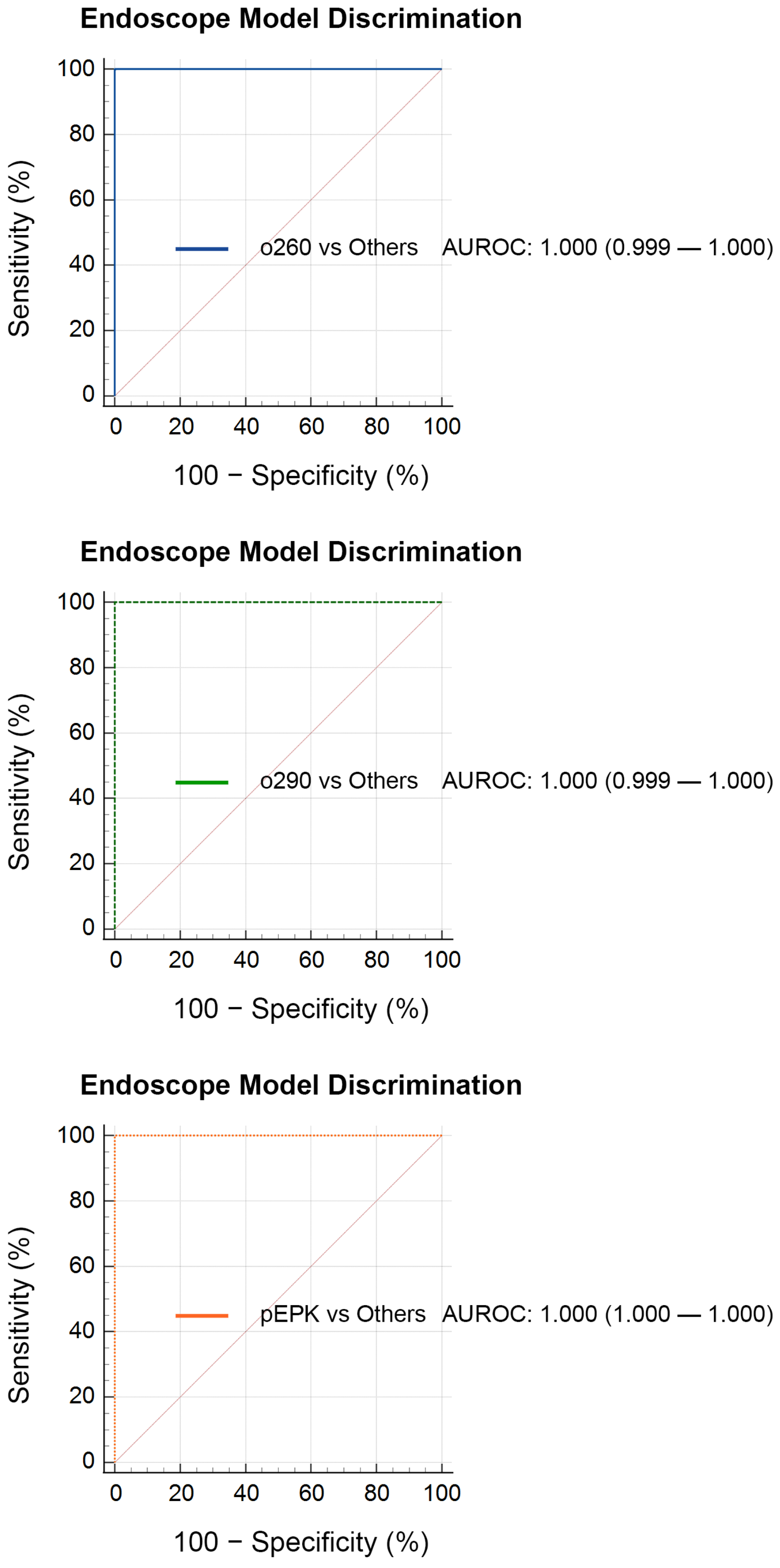

Figure S3. Performance of Endoscope Model Discrimination AI (Experiment 01). All AIs successfully determined the endoscope model that captured images.

o260, Olympus CV-260SL; o290, Olympus CV-290; pEPK, PENTAX EPK-i; AUROC, area under the receiver operating characteristic curve

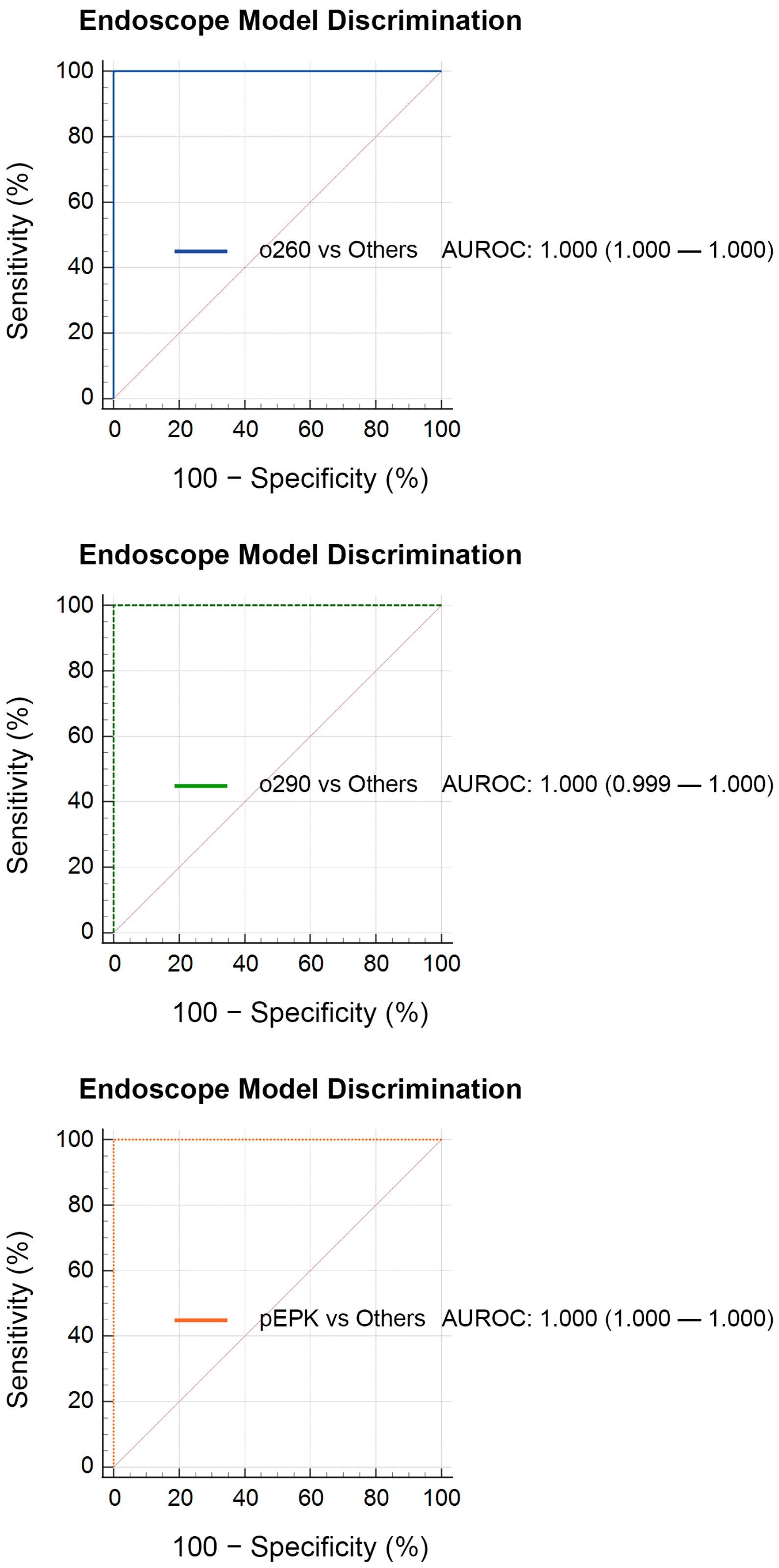

Figure S4. Performance of Endoscope Model Discrimination AI (Experiment 01). All AIs successfully determined the endoscope model that captured images.

o260, Olympus CV-260SL; o290, Olympus CV-290; pEPK, PENTAX EPK-i; AUROC, area under the receiver operating characteristic curve

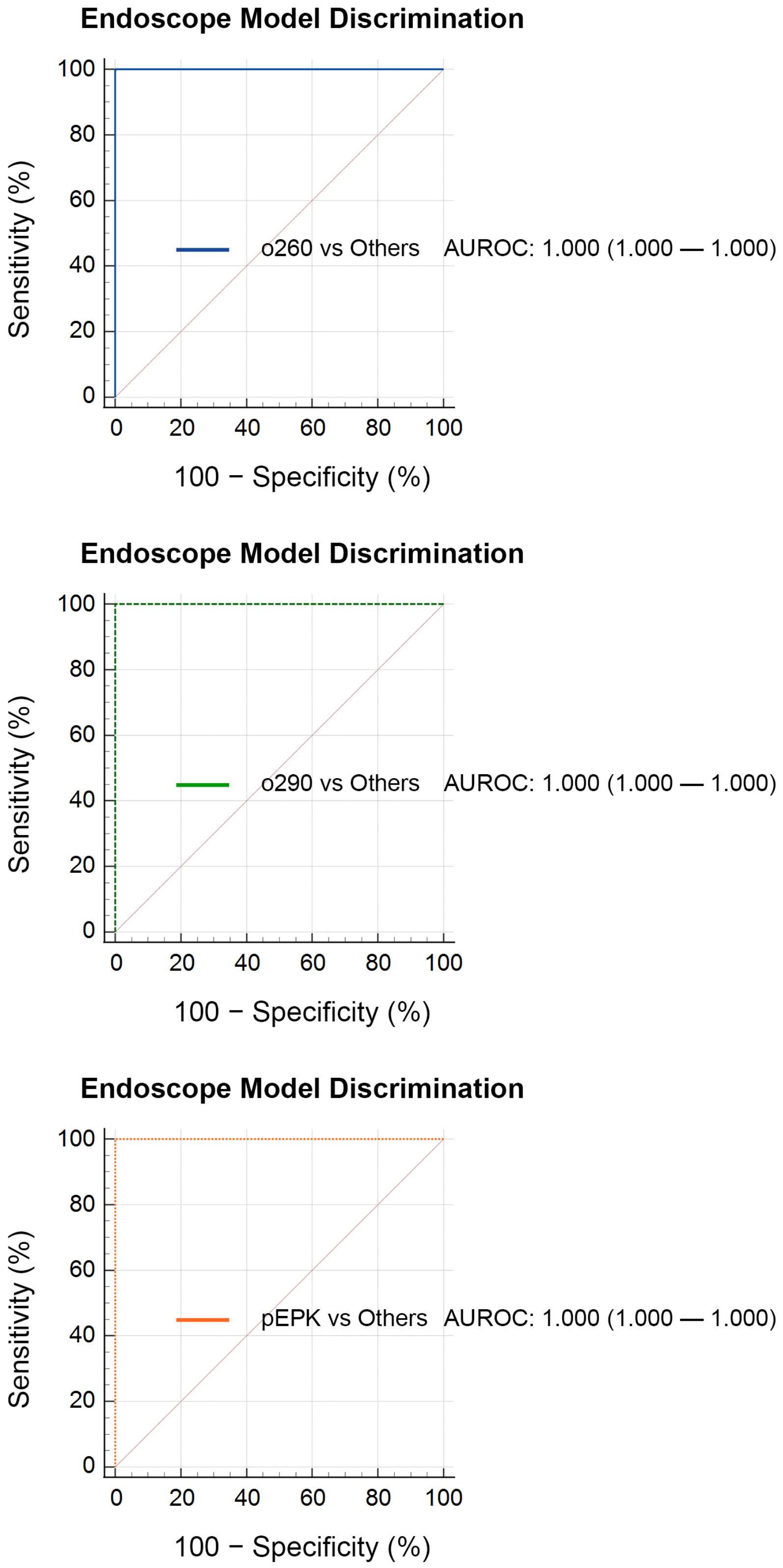

Figure S5. Performance of Endoscope Model Discrimination AI (Experiment 01). All AIs successfully determined the endoscope model that captured images.

o260, Olympus CV-260SL; o290, Olympus CV-290; pEPK, PENTAX EPK-i; AUROC, area under the receiver operating characteristic curve

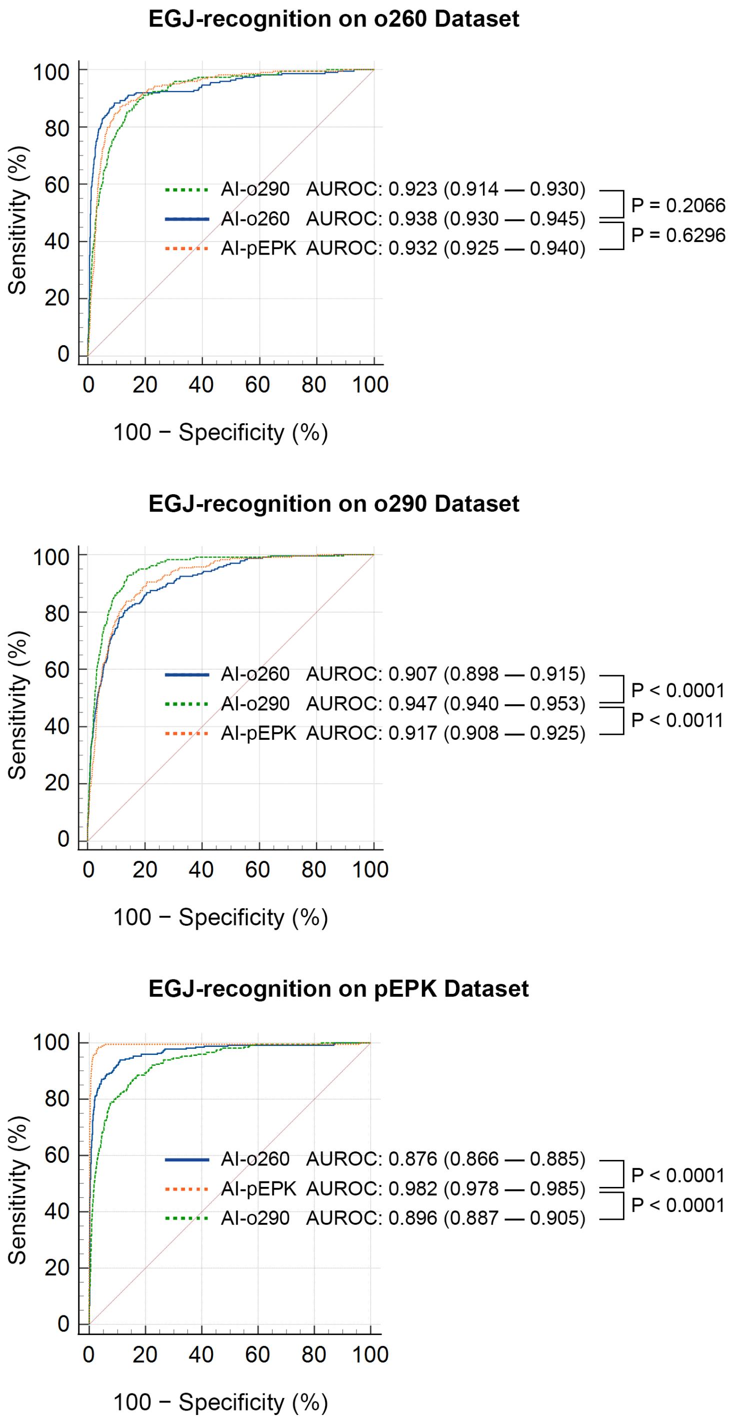

Figure S6. Statistical Comparison of the ROC curves of EGJ-recognition AIs on Validation Datasets. (Experiment 01) EGJ-recognition AIs (AI-o260, AI-o290, and AI-pEPK) trained on a dataset classified by the endoscope model had degraded performance on the validation dataset of other endoscope models.

o260, Olympus CV-260SL; o290, Olympus CV-290; pEPK, PENTAX EPK-i; AI, artificial intelligence; EGJ, esophagogastric junction; AUROC, area under the receiver operating characteristic curve

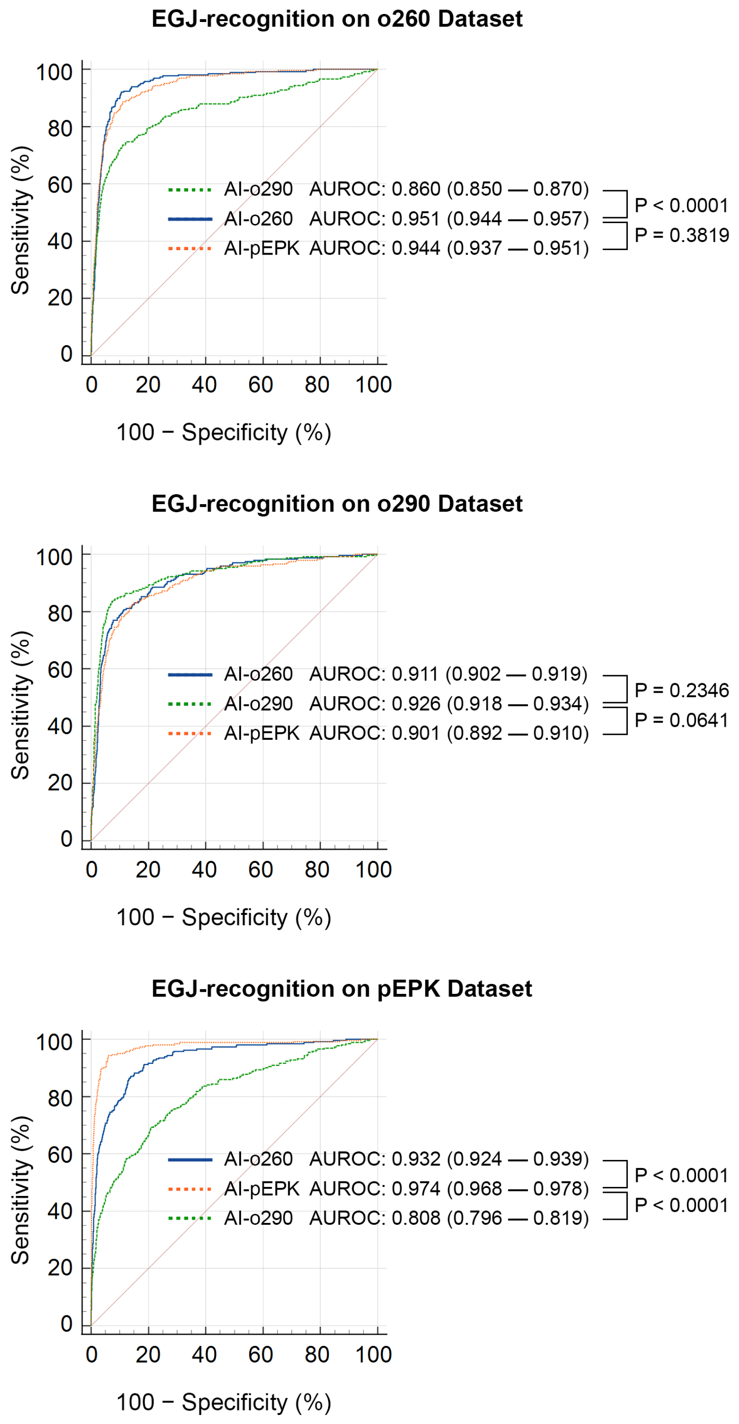

Figure S7. Statistical Comparison of the ROC curves of EGJ-recognition AIs on Validation Datasets. (Experiment 02) EGJ-recognition AIs (AI-o260, AI-o290, and AI-pEPK) trained on a dataset classified by the endoscope model had degraded performance on the validation dataset of other endoscope models.

o260, Olympus CV-260SL; o290, Olympus CV-290; pEPK, PENTAX EPK-i; AI, artificial intelligence; EGJ, esophagogastric junction; AUROC, area under the receiver operating characteristic curve

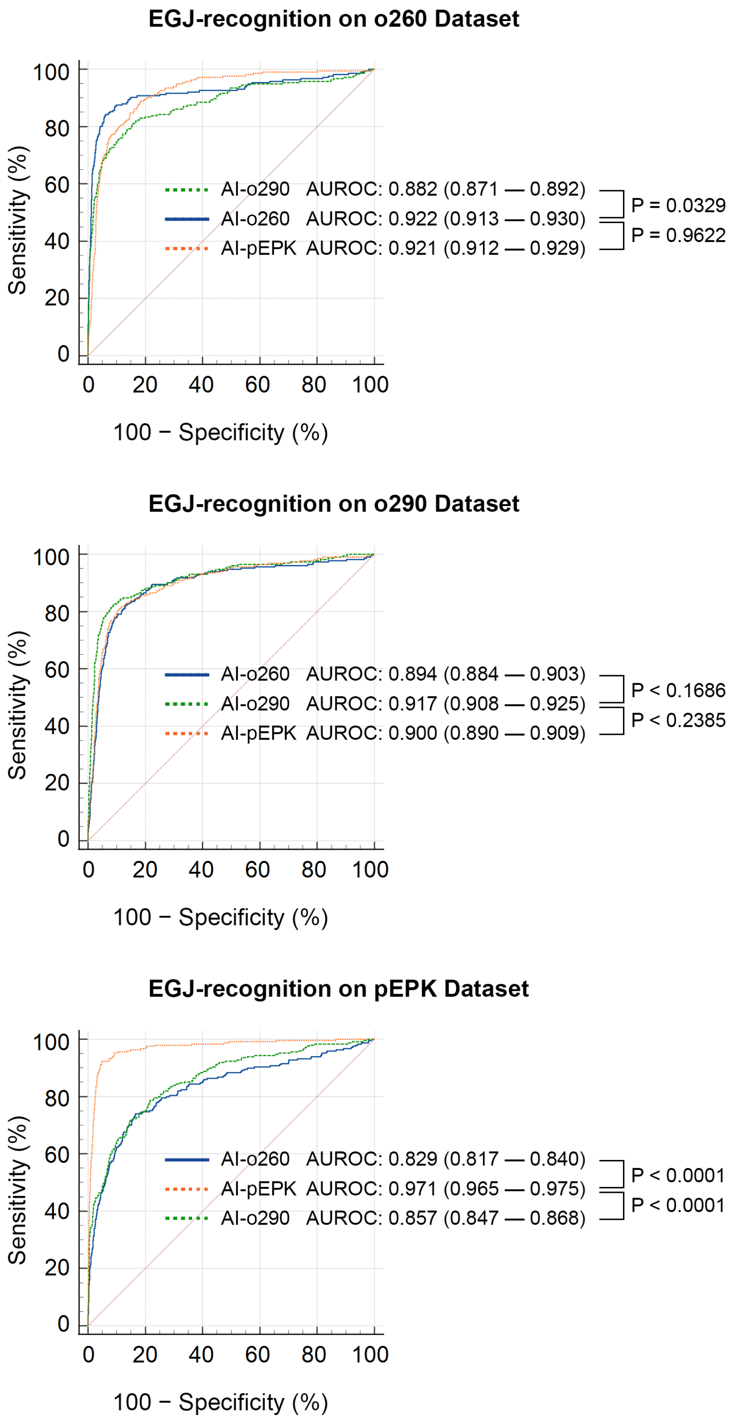

Figure S8. Statistical Comparison of the ROC curves of EGJ-recognition AIs on Validation Datasets. (Experiment 03) EGJ-recognition AIs (AI-o260, AI-o290, and AI-pEPK) trained on a dataset classified by the endoscope model had degraded performance on the validation dataset of other endoscope models.

o260, Olympus CV-260SL; o290, Olympus CV-290; pEPK, PENTAX EPK-i; AI, artificial intelligence; EGJ, esophagogastric junction; AUROC, area under the receiver operating characteristic curve

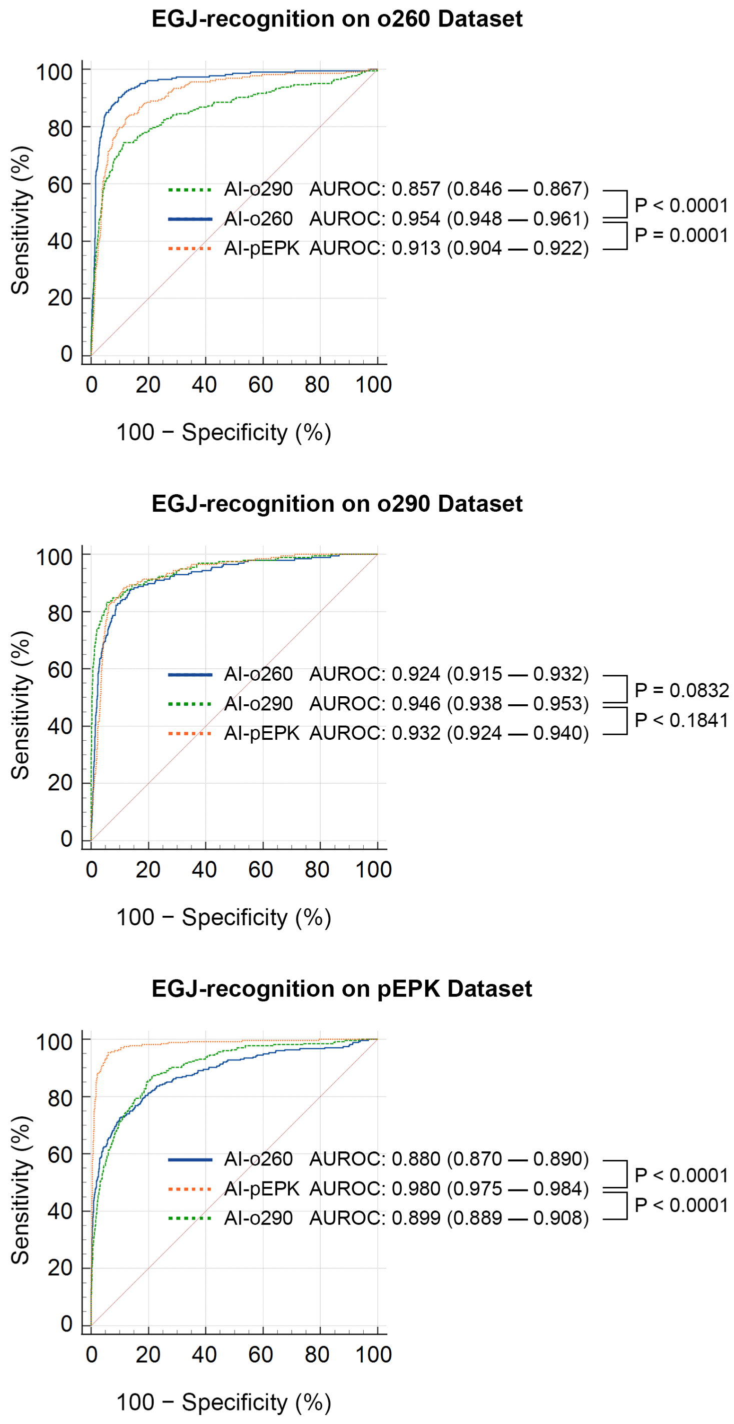

Figure S9. Statistical Comparison of the ROC curves of EGJ-recognition AIs on Validation Datasets. (Experiment 04) EGJ-recognition AIs (AI-o260, AI-o290, and AI-pEPK) trained on a dataset classified by the endoscope model had degraded performance on the validation dataset of other endoscope models.

o260, Olympus CV-260SL; o290, Olympus CV-290; pEPK, PENTAX EPK-i; AI, artificial intelligence; EGJ, esophagogastric junction; AUROC, area under the receiver operating characteristic curve

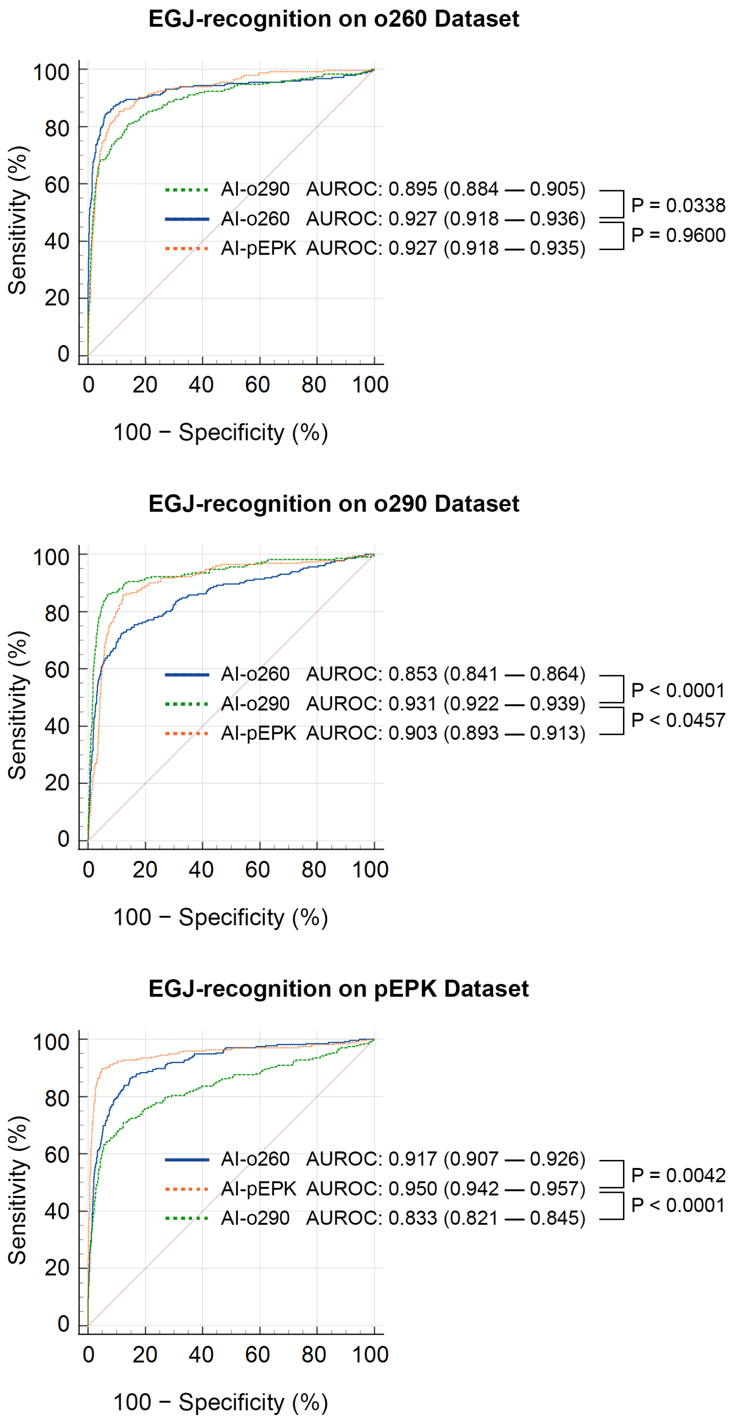

Figure S10. Statistical Comparison of the ROC curves of EGJ-recognition AIs on Validation Datasets. (Experiment 05) EGJ-recognition AIs (AI-o260, AI-o290, and AI-pEPK) trained on a dataset classified by the endoscope model had degraded performance on the validation dataset of other endoscope models.

o260, Olympus CV-260SL; o290, Olympus CV-290; pEPK, PENTAX EPK-i; AI, artificial intelligence; EGJ, esophagogastric junction; AUROC, area under the receiver operating characteristic curve
