# Supplementary figures and images for: Reduced detection rate of artificial intelligence in images obtained from untrained endoscope models and improvement using domain adaptation algorithm
Source: Front Med (Lausanne). 2022 Nov 10;9:1036974. doi: 10.3389/fmed.2022.1036974 (PMC9684642; doi:10.3389/fmed.2022.1036974)

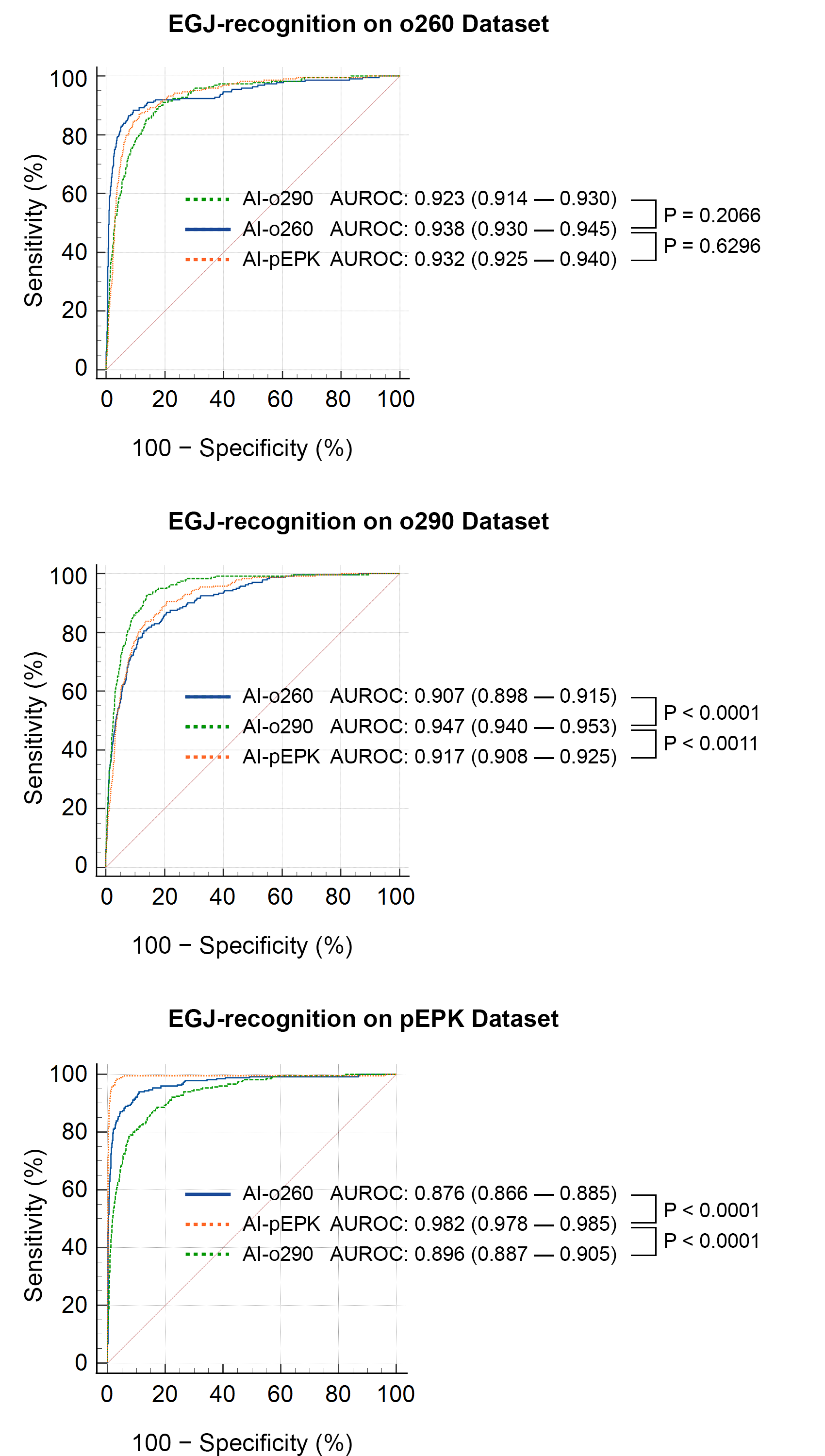

Supplement: Supplementary file 2 [file Image_1.TIF]

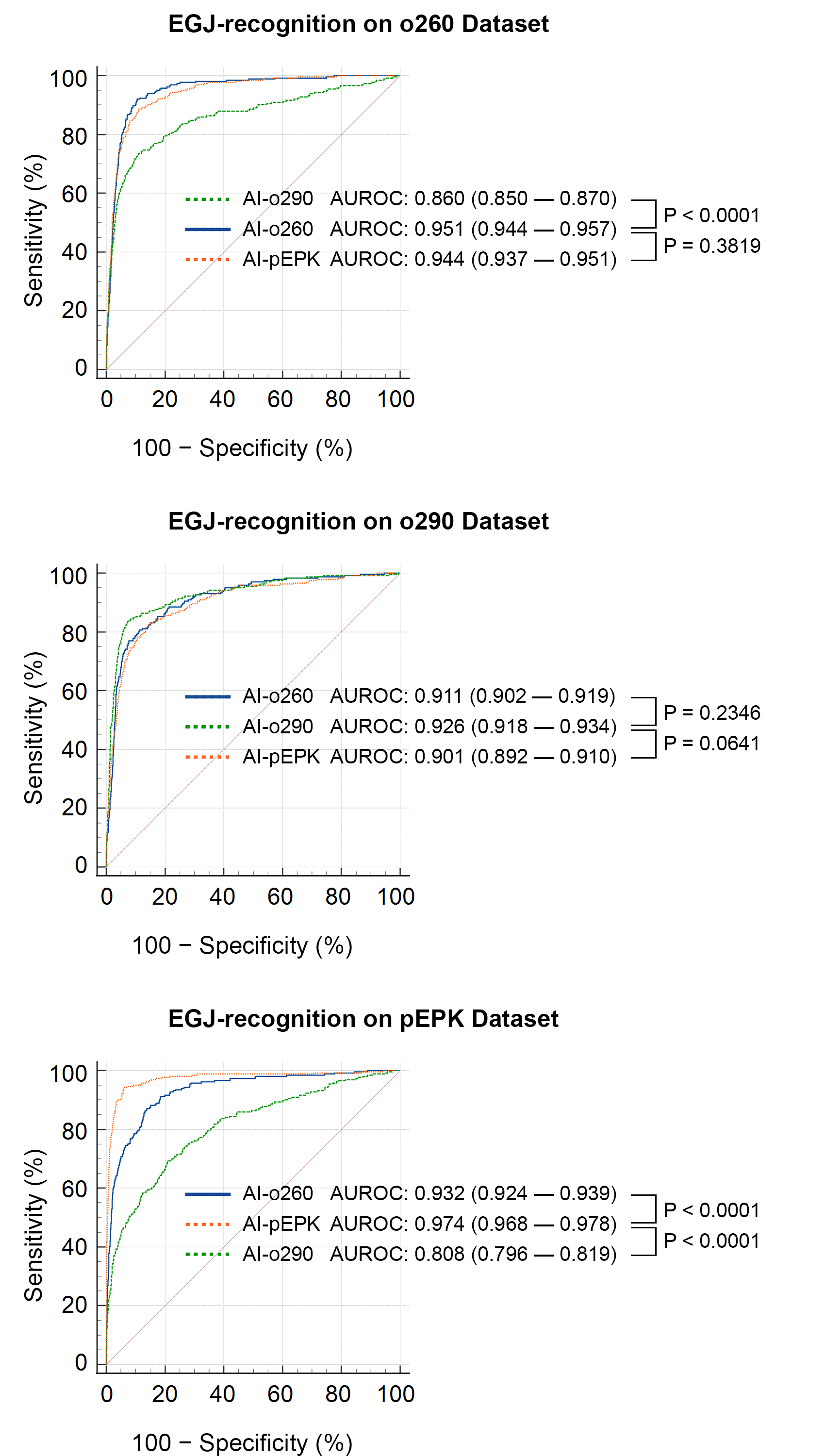

Supplement: Supplementary file 3 [file Image_2.TIF]

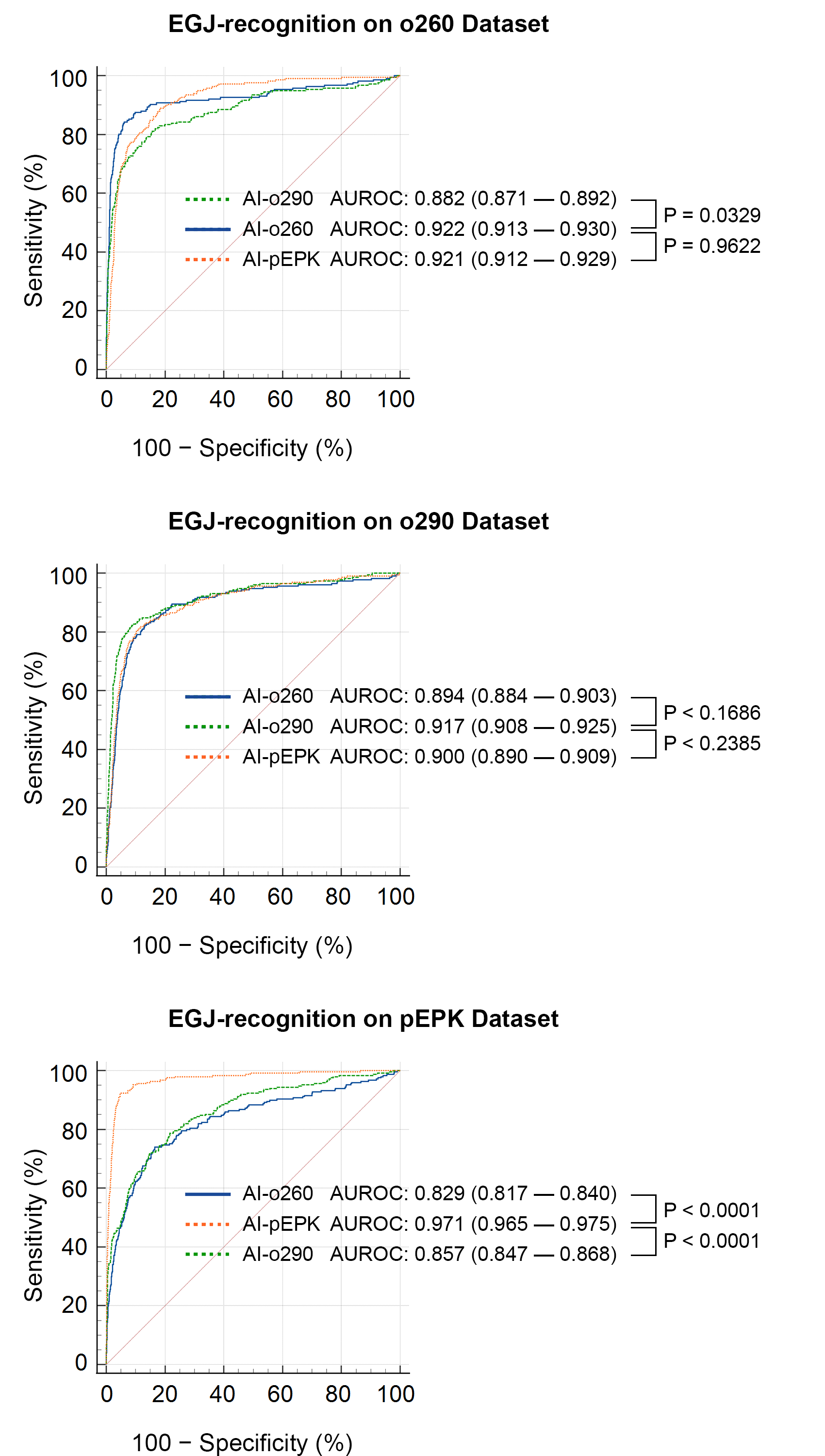

Supplement: Supplementary file 4 [file Image_3.TIF]

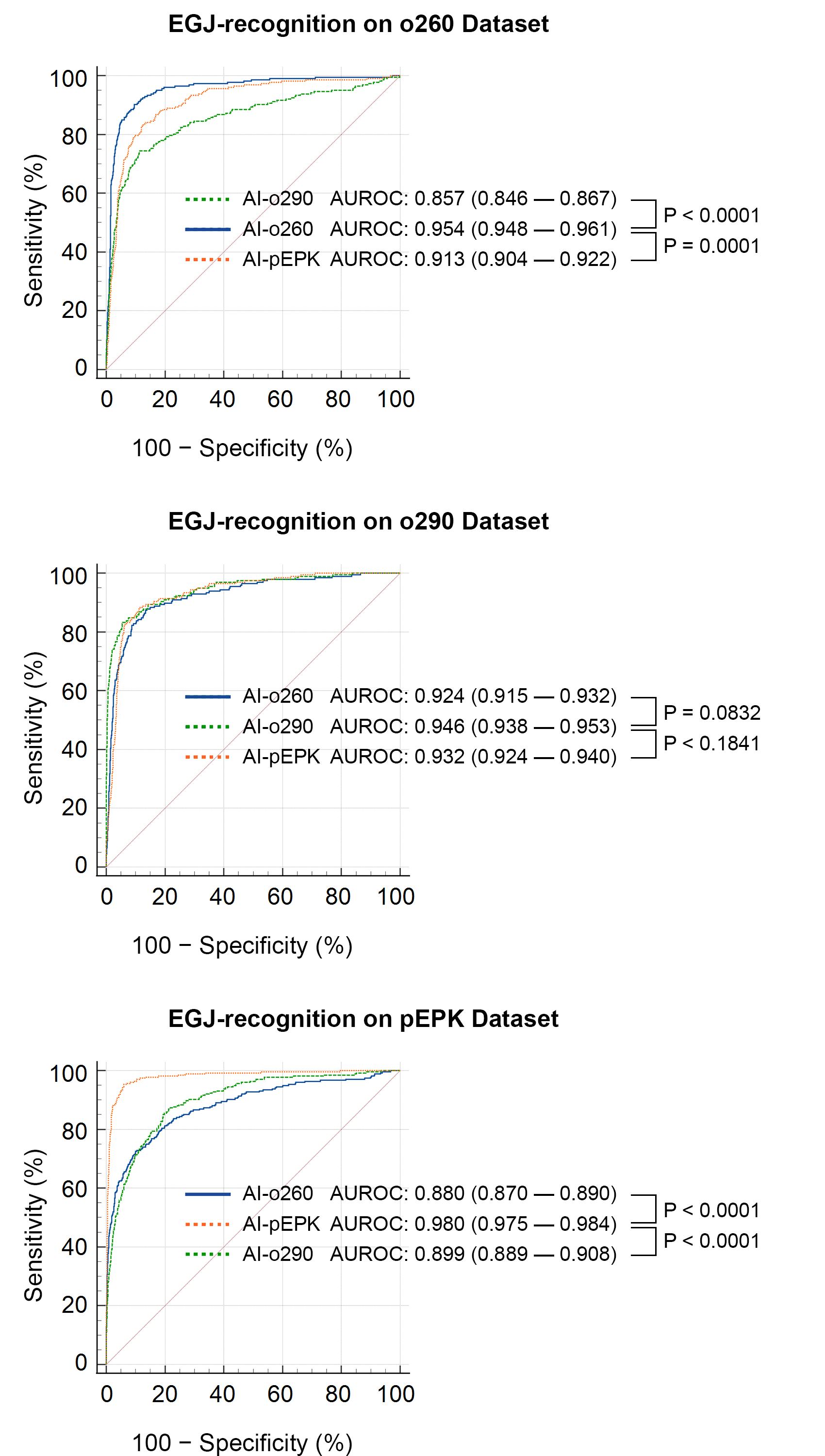

Supplement: Supplementary file 5 [file Image_4.TIF]

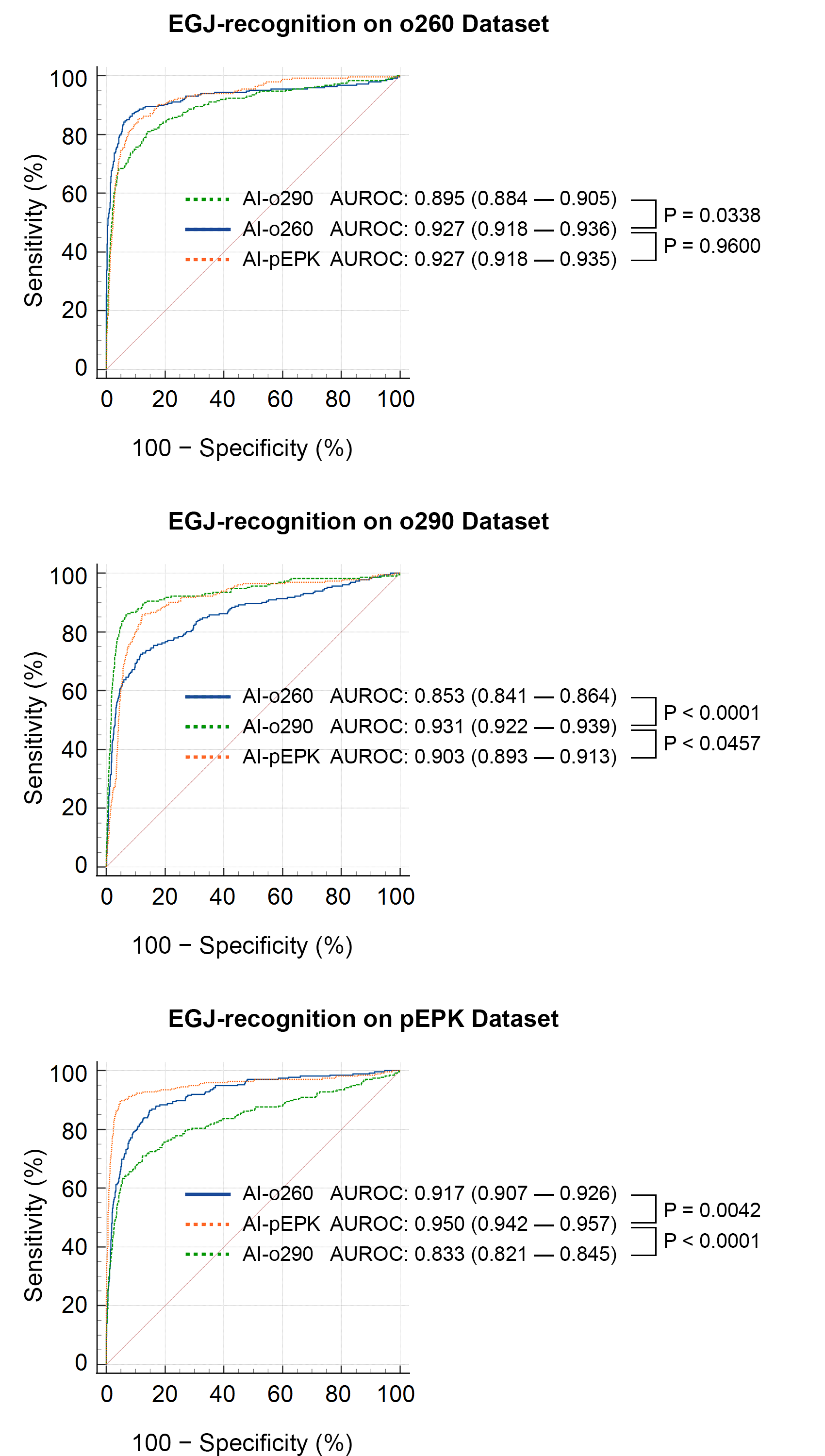

Supplement: Supplementary file 6 [file Image_5.TIF]

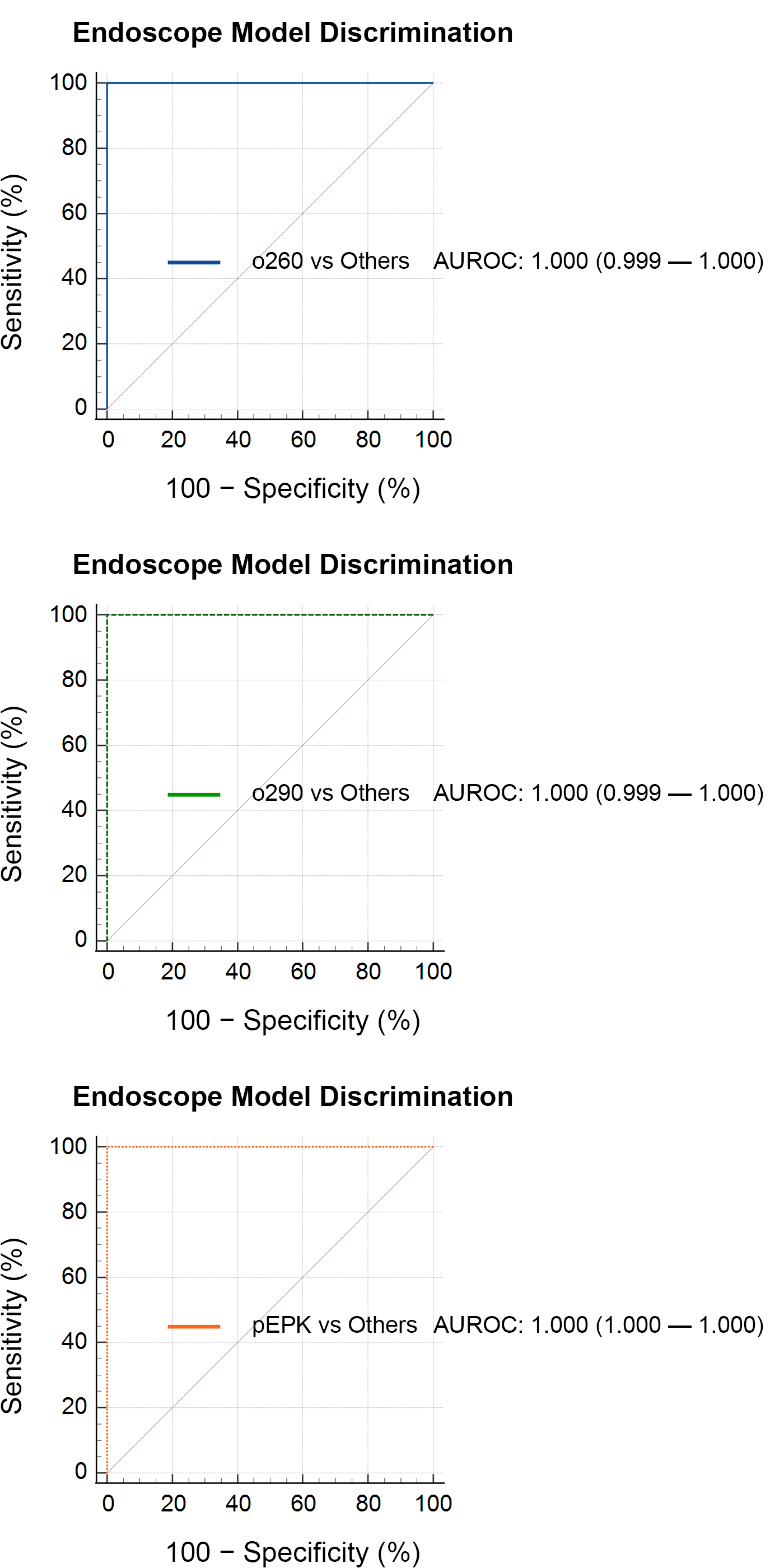

Supplement: Supplementary file 7 [file Image_6.TIF]

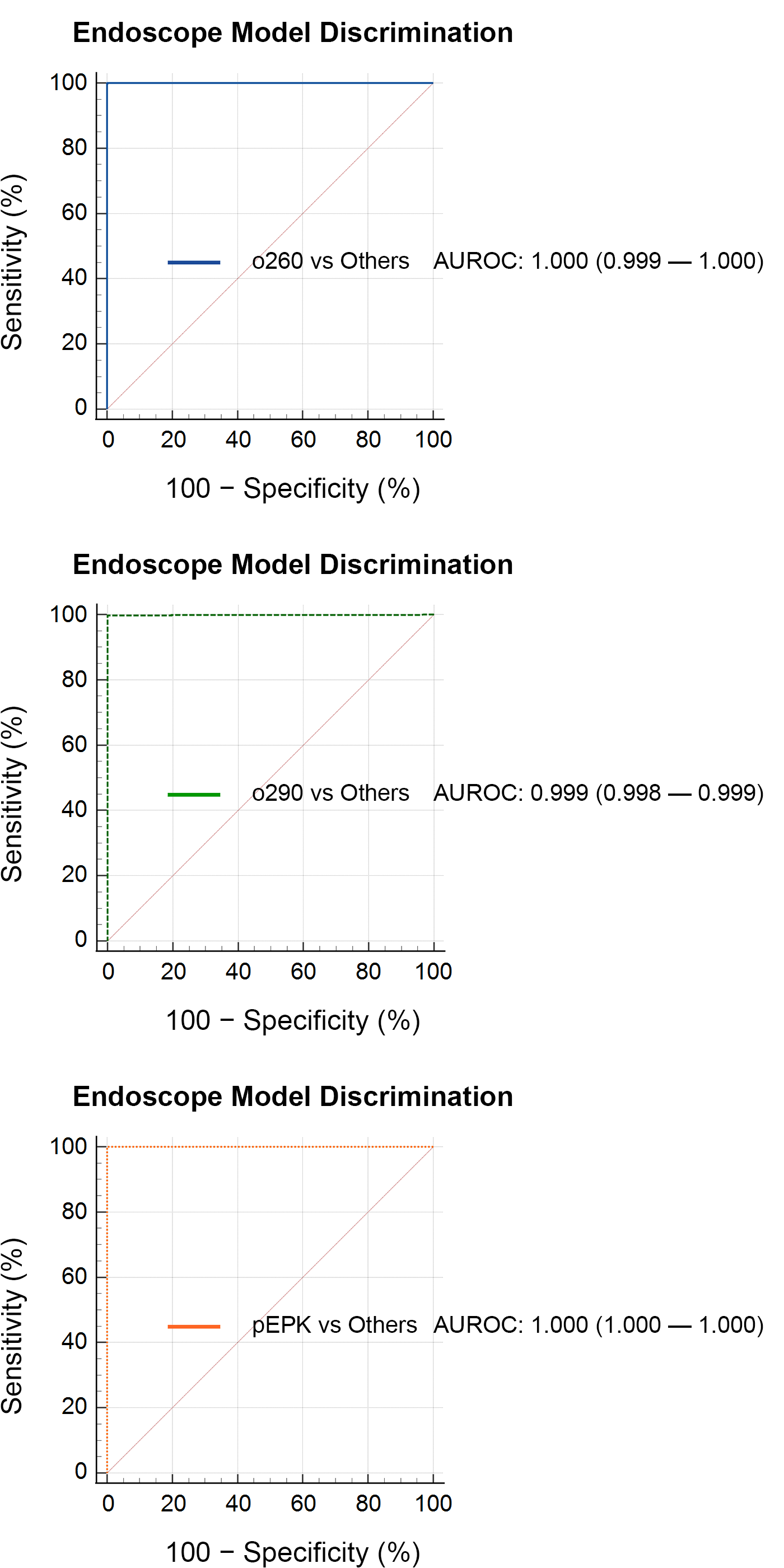

Supplement: Supplementary file 8 [file Image_7.TIF]

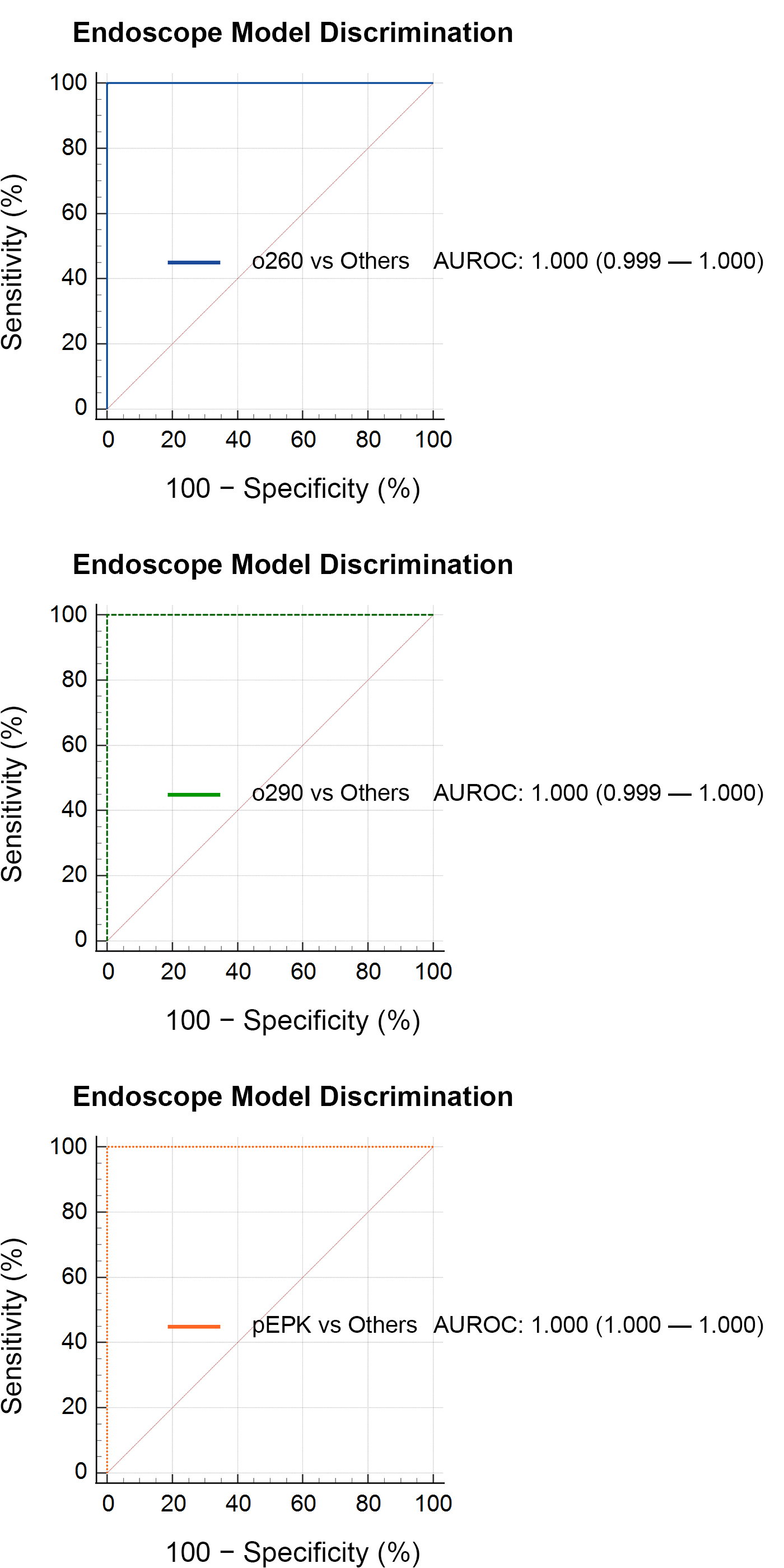

Supplement: Supplementary file 9 [file Image_8.TIF]

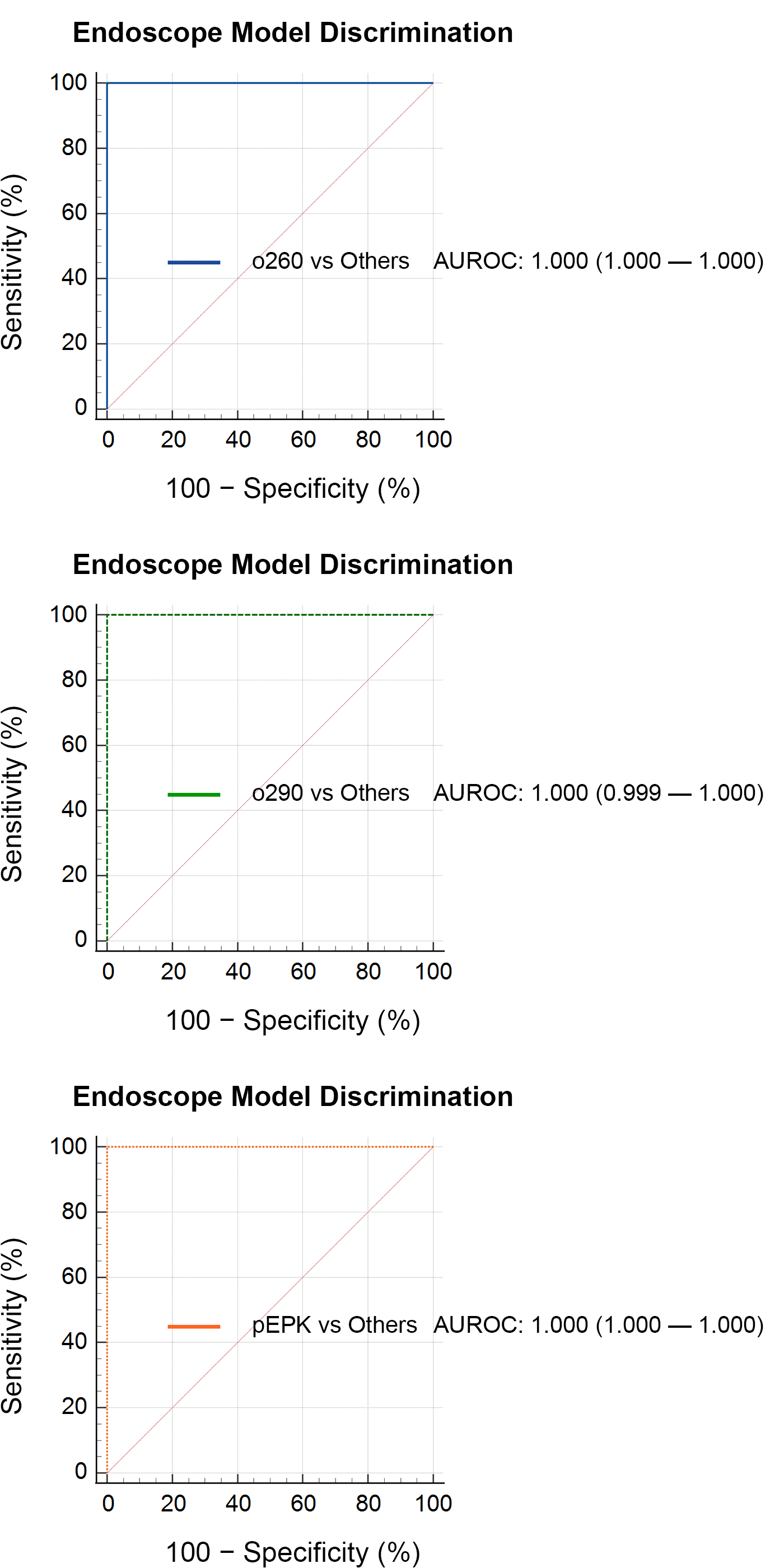

Supplement: Supplementary file 10 [file Image_9.TIF]

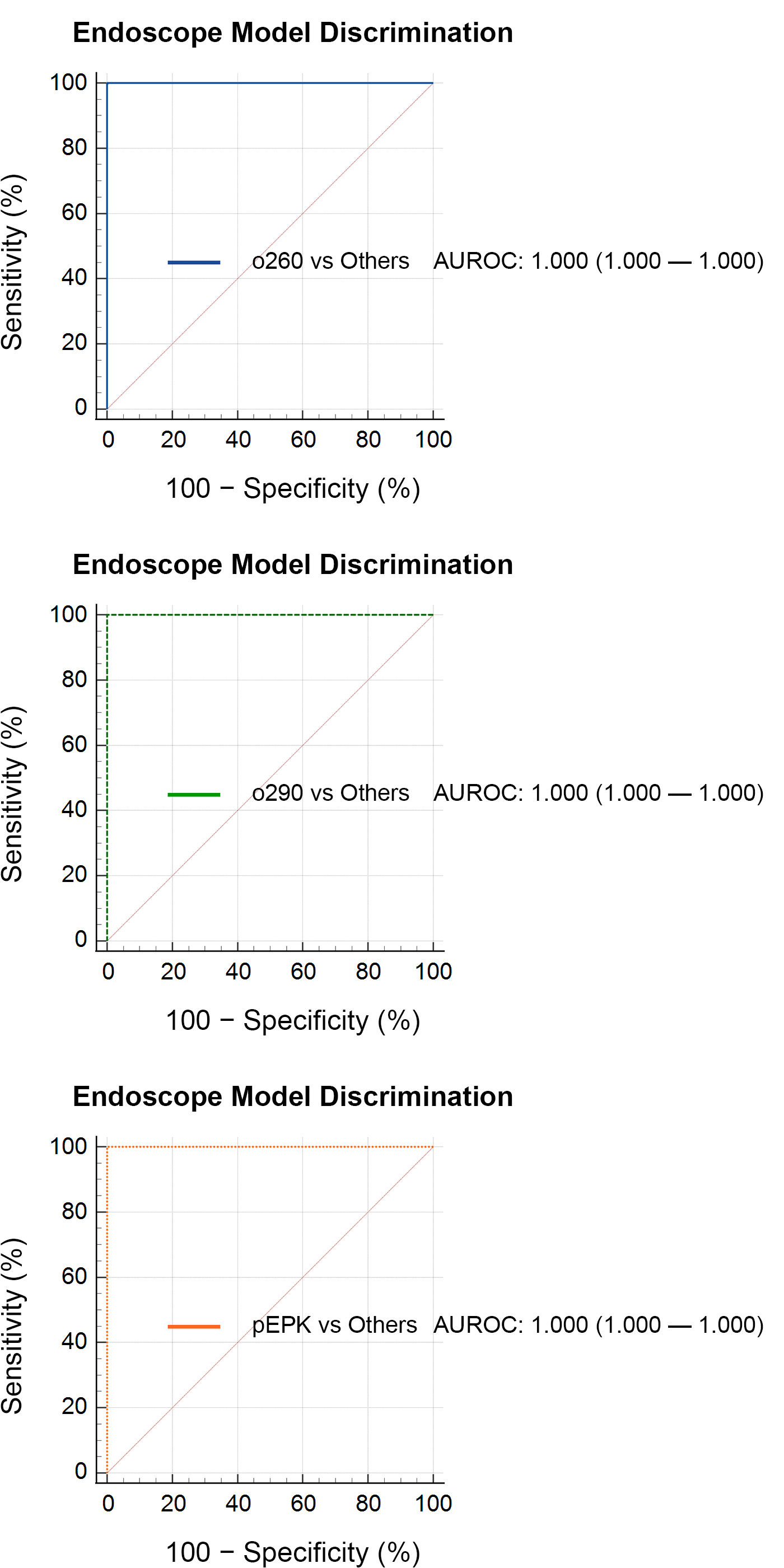

Supplement: Supplementary file 11 [file Image_10.TIF]
